# Systems Modelling of the Socio-Technical Aspects of Residential Electricity Use and Network Peak Demand

**DOI:** 10.1371/journal.pone.0134086

**Published:** 2015-07-30

**Authors:** Jim Lewis, Kerrie Mengersen, Laurie Buys, Desley Vine, John Bell, Peter Morris, Gerard Ledwich

**Affiliations:** 1 Science and Engineering Faculty, Queensland University of Technology (QUT), Brisbane, Queensland, Australia; 2 Creative Industries Faculty, Queensland University of Technology (QUT), Brisbane, Queensland, Australia; Newcastle University, UNITED KINGDOM

## Abstract

Provision of network infrastructure to meet rising network peak demand is increasing the cost of electricity. Addressing this demand is a major imperative for Australian electricity agencies. The network peak demand model reported in this paper provides a quantified decision support tool and a means of understanding the key influences and impacts on network peak demand. An investigation of the system factors impacting residential consumers’ peak demand for electricity was undertaken in Queensland, Australia. Technical factors, such as the customers’ location, housing construction and appliances, were combined with social factors, such as household demographics, culture, trust and knowledge, and Change Management Options (CMOs) such as tariffs, price, managed supply, etc., in a conceptual ‘map’ of the system. A Bayesian network was used to quantify the model and provide insights into the major influential factors and their interactions. The model was also used to examine the reduction in network peak demand with different market-based and government interventions in various customer locations of interest and investigate the relative importance of instituting programs that build trust and knowledge through well designed customer-industry engagement activities. The Bayesian network was implemented via a spreadsheet with a tickbox interface. The model combined available data from industry-specific and public sources with relevant expert opinion. The results revealed that the most effective intervention strategies involve combining particular CMOs with associated education and engagement activities. The model demonstrated the importance of designing interventions that take into account the interactions of the various elements of the socio-technical system. The options that provided the greatest impact on peak demand were Off-Peak Tariffs and Managed Supply and increases in the price of electricity. The impact in peak demand reduction differed for each of the locations and highlighted that household numbers, demographics as well as the different climates were significant factors. It presented possible network peak demand reductions which would delay any upgrade of networks, resulting in savings for Queensland utilities and ultimately for households. The use of this systems approach using Bayesian networks to assist the management of peak demand in different modelled locations in Queensland provided insights about the most important elements in the system and the intervention strategies that could be tailored to the targeted customer segments.

## Introduction

This paper investigates a quantified method of integrating the social and technical factors involved in network peak demand for electricity in residential households. Globally, meeting the rapid increase in network peak demand for electricity is a significant challenge for electricity utilities. Billions of dollars are needed to update transmission, distribution and generation infrastructure to guarantee electricity supply during network peak periods for only a handful of days per year [1, 2]. The Queensland Government estimated that distributors would need to spend approximately $5000 for each additional megawatt (MW) of network peak energy consumption [3]. This rapidly increasing capital investment in electricity provision requires rethinking the traditional model of building supply to meet demand [2], and finding cost effective ways to reduce peak demand is a major imperative for electricity utilities.

Network peak demand is a complex system [4] in which demand management provides a means of finding solutions that assist electricity utilities to ensure they have sufficient network capacity to meet peak demand, thereby helping to avoid or delay network upgrades that would otherwise be required. Residential use of electricity, defined as residential loads, contributes significantly to seasonal and daily peak electricity demand [5] and accounts for approximately one third of the total peak electricity demand [6]. There is thus a strong interest in encouraging residential consumers to change their electricity demand patterns at times of network peaks [7].

Since the 1970s, there have been a plethora of frameworks, theories and interventions aimed at changing the behaviour of residential electricity consumers. These have arisen from various disciplines, including economics, engineering, sociology, anthropology and psychology, but they do not provide a reliable, quantified predictive tool for intervention [8–13]. The supply and demand of electricity exists within a very complex system with many interacting elements that cannot be reduced to simple explanations or policy approaches [14]. Residential electricity demand is influenced by behaviour of consumers as well as by the physical environment, house construction, and acquired and available appliances, other infrastructure, price, industry and government policies, incentives, interventions and so on. In order to address the multifaceted challenges of this complex system and achieve more realistic and wide-ranging understanding of residential energy consumption, there has been a growing call for integrated approaches to analysis rather than those provided by single disciplinary studies [8, 10, 11, 15, 16].

An approach that is gaining substantial momentum in many areas is to accommodate the complexity of the system, and the various sources of information that inform it, in a dedicated systems model, such as a Bayesian network (BN) [17–21]. BNs are statistical models that provide a graphical, probabilistic framework for representing and analysing domains involving uncertainty [22]. They facilitate the integration of information from diverse sources, including data, other literature and expert judgement, using a transparent, efficient and mathematically rigorous process [23–25]. A BN is typically constructed in two stages: first, a conceptual ‘map’ of the system is developed, whereby the target outcome and the suite of factors that potentially affect the target are represented by nodes (circles) and the linkages or interactions between these nodes are represented by arrows. The conceptual map is then quantified using a suite of probability tables or distributions based on the available information. The BN can then be used to examine scenarios, identify the most important factors impacting on the target, highlight knowledge or information gaps, evaluate the impact of changes in the system and, suggest strategies for obtaining optimal outcomes [23].

**Fig 1:**
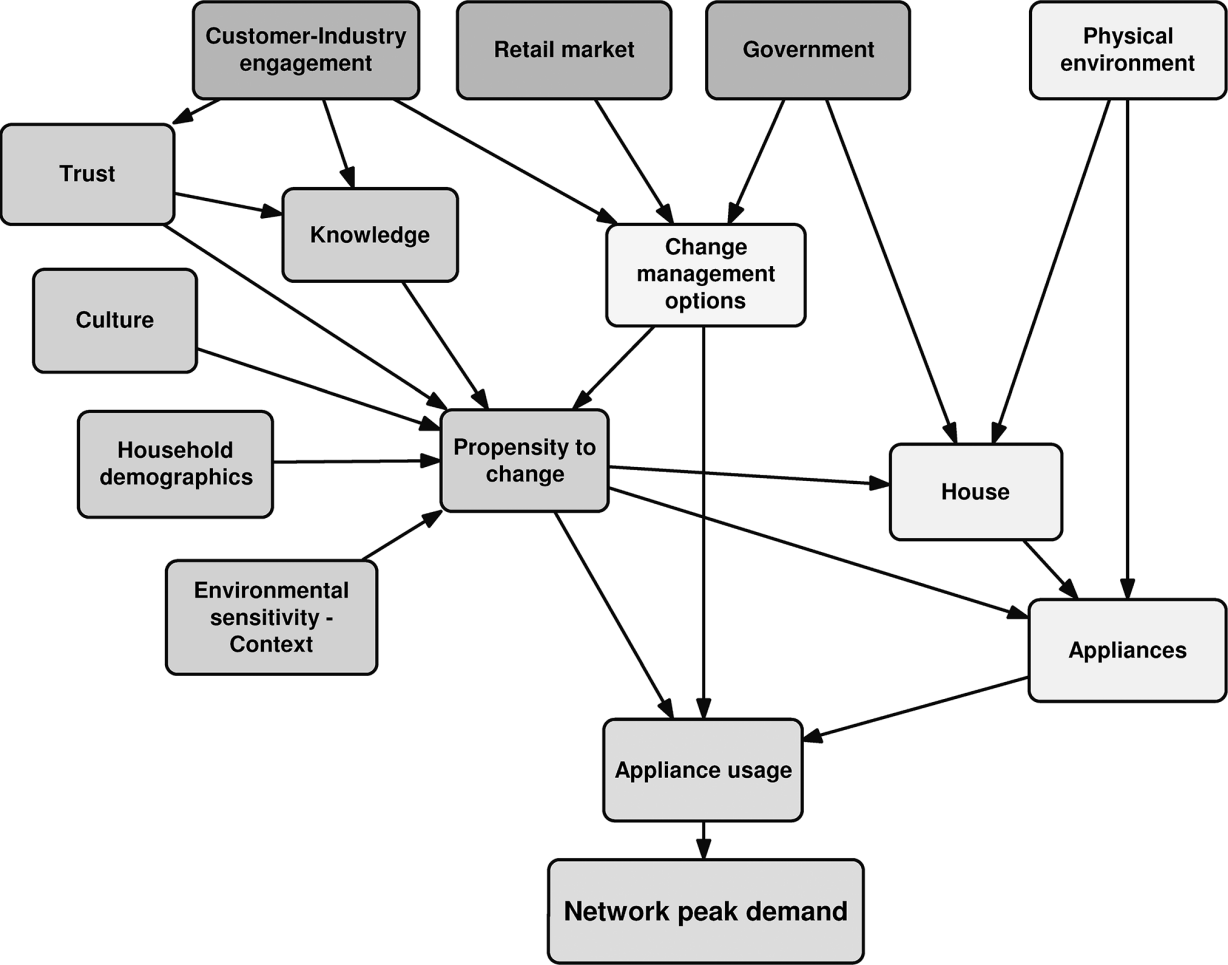
Residential electricity peak demand model.

This paper reports on a method of quantifying a recently developed model by Buys and colleagues [26], Residential Electricity Peak Demand Model (REPDM), that used this integrative approach to address residential energy demand reduction at peak times, see Fig 1. The model combines the socio-technical aspects of residential demand and investigates this complexity through modelling the impact of the probabilistic responses and connections between the social elements and the technical aspects of the system. It provides a decision support tool and a means of understanding the key influences and impacts on network peak demand as the model allows various intervention scenarios to be tested and these give insights into how the system could be managed. The purpose of this paper is to examining the reduction in network peak demand, in a quantified model, with scenarios of possible interventions in the socio-technical system.

## Method

This research was part of a larger study looking at electricity demand-side management: Models, optimisation and customer engagement. The aim was to develop an integrated, quantified model of the socio-technical aspects of interventions that would reduce network peak demand by residential consumers.

The university and industry members of the project research team provided their verbal consent to give their professional opinion in the development of the model and this consent was documented. The research team held regular team meetings using a workshop format and obtained data from available secondary sources or from their own industry or professional expert knowledge (as outlined in the paper). No individually identifiable/participant level data were collected for this study.

### Structured development of a systems model

**Fig 2:**
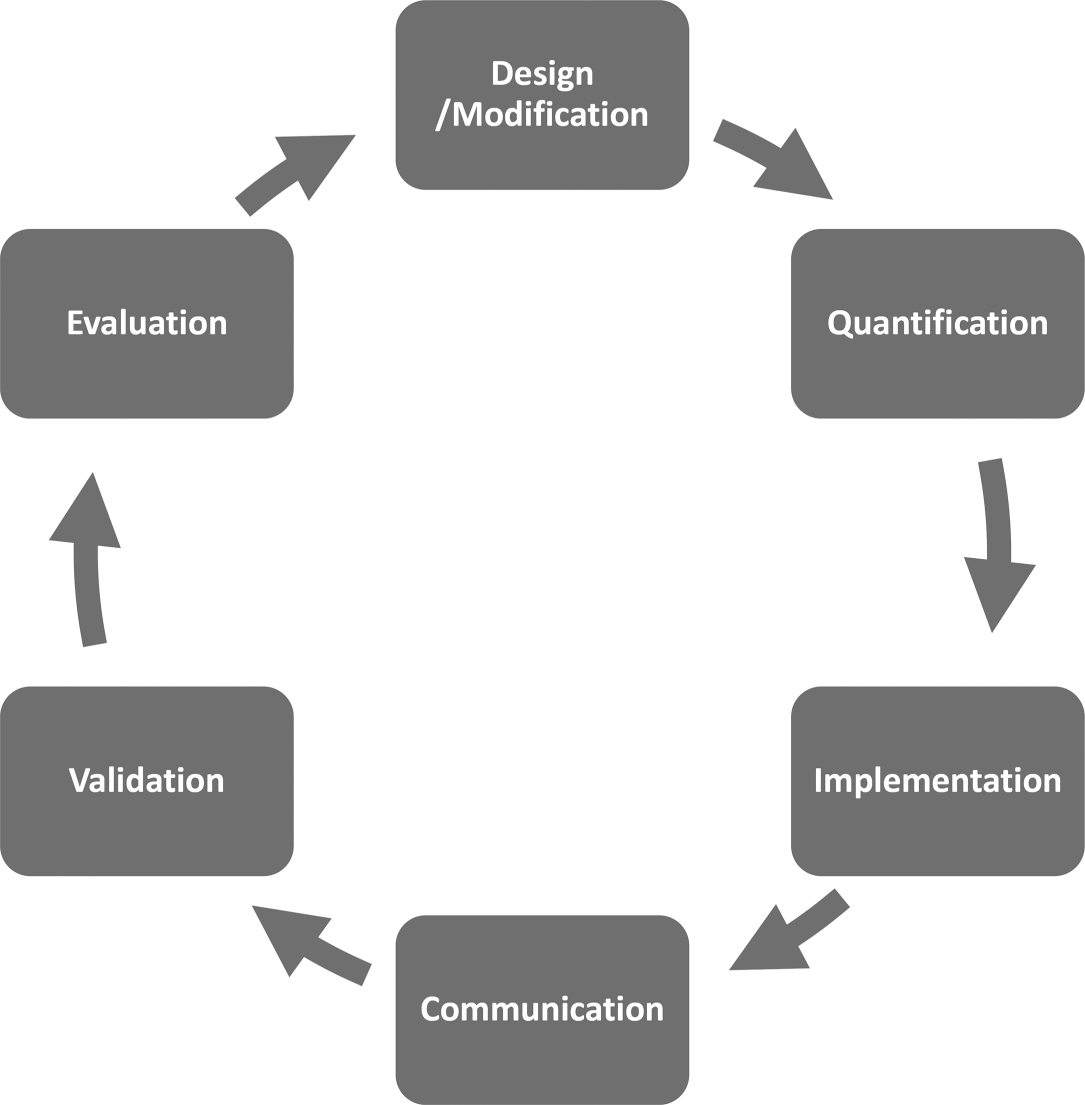
Model development cycle.

The components of the structured development process (Fig 2), namely Design, Quantification, Implementation, Communication, Validation and Evaluation for the REPDM are described in the following sections. The model development was an interactive, participative process involving structured workshops and meetings with working groups consisting of industry and academic project team members. The participative process was used both to elicit the parameters of the model and to gain information about information gaps. The structure of the model was validated as part of this process both in the development of the REPDM and in its implementation through a review process with the working groups. The validation of the BN model aimed at checking the Excel implementation, the states of the nodes of the model and the outputs.

**Fig 3:**
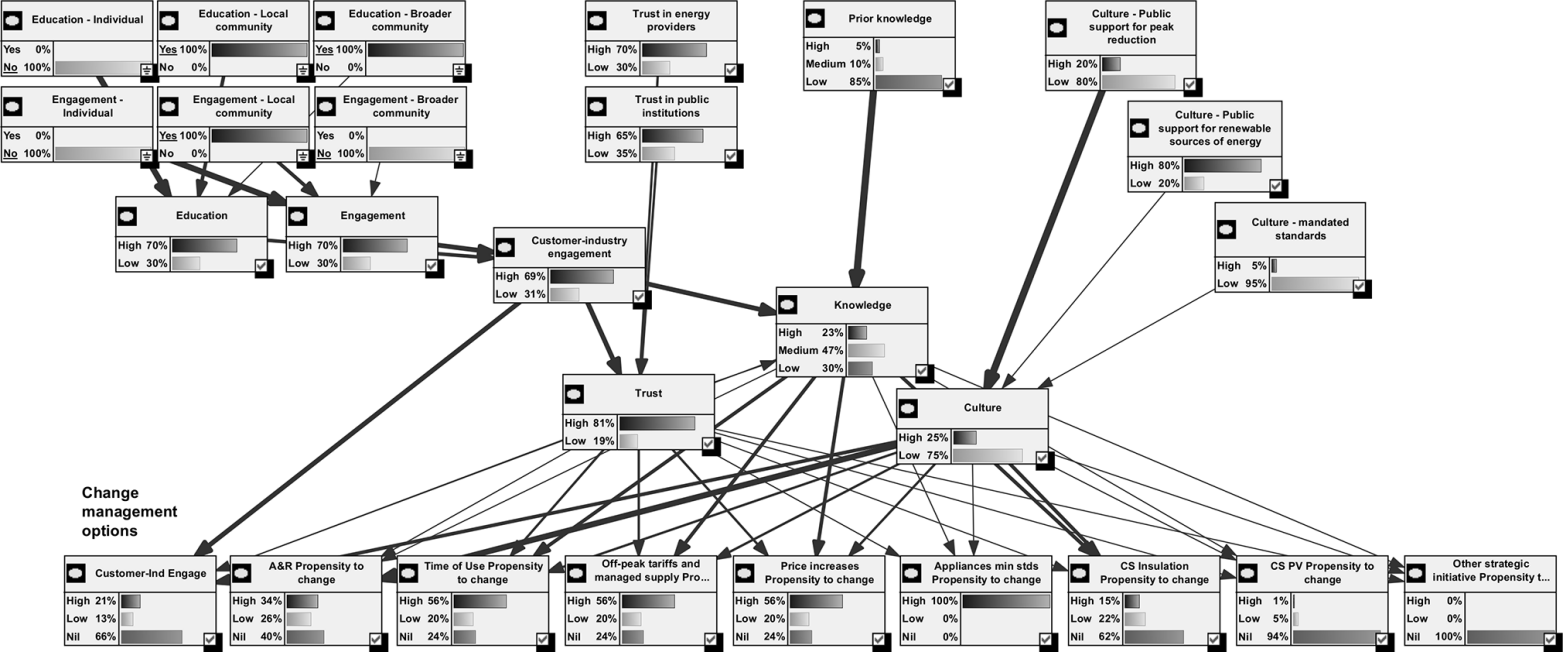
BN of social dimensions and Strength of influence.

The BN was developed by taking the elements and links in the conceptual model (Fig 1) and translating these into nodes and edges of a BN graphical model. In the BN, probability distributions for a node are influenced only by those nodes with directed arrows feeding into the given node in the system. Some of the elements were further refined into sub-networks (Fig 3).

Although presented as a cycle, the development process depicted in Fig 2 does not necessarily proceed sequentially and can be modified according to the demands of the problem and/or the corresponding system being developed. For example, implementation and quantification may occur in reverse order or in parallel, and similarly validation and communication may be combined.

#### Design

As described by Buys and colleagues [26], the design phase involved engaging stakeholders to design the conceptual model, including identifying nodes and the interactions between the nodes. The conceptual design of the network demand systems model was undertaken by a multidisciplinary team of researchers and industry experts through a series of eight intensive project team workshops and 15 subgroup workshops.

The REPDM (Fig 1) combines the social dimensions of energy use (including knowledge, trust, culture, household demographics, propensity to change and environmental sensitivity (context)), and the technical elements (including physical environment, house, appliances) related to the house, the type of appliances that may be used and the physical environment (location). The stakeholders determining policy, marketing and CMOs were the government and the retail market in interaction with customers.

In Fig 1, the nodes are represented as single terms, but are in fact the sub-networks that describe sets of factors that affect these higher level nodes. The sub-networks for some of the nodes are presented in Fig 3 and are described in more detail in Supporting Information (S2 PDF and S1, S2 and S3 Tables). Detailed descriptions of the nodes and other terms used in building the model ensured uniform understanding of the terms and a consistent and repeatable quantification of items.

The model was designed to accommodate interactions between the nodes. For example, the retail market and government policy elements can interact with the electricity customers to explore possible change management outcomes. Although the Change Management Option node and the Propensity to Change node are represented as a single link in Fig 1, in the BN model each of the options is included as a separate node within the BN structure. The Propensity to Change for each Change Management Option is combined in the Appliance usage node to give the network peak demand.

#### The Change Management Options

The choice of CMOs to be modelled were drawn from an internal report of the industry partner [27]. The report identified a number of interventions and also specified the target demographics for each intervention. The electricity use by each segment was used as a proxy for the network peak electricity use by the targeted segment group. The CMOs are described in more details in the Supplementary information (S6 Table).

#### Quantification

As described above, quantification of the conceptual model was achieved by developing the model as a BN and using available data to develop conditional probability distributions as well as quantify the relative influences of the nodes of the model. The probabilities were derived from industry reports and surveys, literature search and workshops with industry partners and academic experts (See Table 1). These data were taken as the initial estimates for the values to be modelled to allow the value of the approach to be investigated. These probabilities were represented as conditional probability tables (CPTs) with the states of each child node being defined by the states of each of its parent nodes.

**Table 1:** Information sources used to quantify the BN.

System element (node)	Data type	Source
Knowledge	Customer surveys, industry workshops	Internal industry data and reports
Trust	Customer surveys and workshop	Internal industry data and reports
Culture	Industry partner workshops	Internal industry data and reports
Household demographics	Customer survey	Ergon Energy [27]
Change Management Options	Industry report	Ergon Energy [27]
CPTs for Trust, Knowledge, Culture, Propensity to Change, Appliances, CMOs	Industry partner workshops	Refereed research, industry social marketing research, and industry and academic experts
Environmental sensitivity.	Industry partner workshops	Refereed research, industry social marketing research, and industry and academic experts
Appliances	Industry partner data and workshops	Refereed research, and industry and academic experts
Physical environment (including number of households)	Publicly available, industry partner workshops	Household numbers from ABS census data, Acxiom PersonicX demographic categories, and industry experts.
House (heat load)	Modelling head load	Modelling by academic and industry experts
Retail market, Government policy and Customer/Industry engagement	Change Management Options	Ergon Energy [27]

Where possible the CPTs were quantified using available data. CPTs derived from expert judgement were developed through a structured expert elicitation process [28] with the assistance of a CPT Calculator [29]. The calculator reduces the number of probabilities to be elicited to key state combinations of parent nodes in order to enhance the consistency of the elicited values and the efficiency of the elicitation process.

The development of the CPTs relating to the social factors was undertaken in workshops with industry experts who also had access to internal social marketing research that assisted with the quantification of the probability states. The elicitation process involved the development and discussion of focus questions to ensure that a shared and consistent understanding of the impact of the input nodes on the probability states of the outputs. As part of the sequenced workshop process, the completed CPTs were provided to the industry partners for comment and feedback.

As described above, the BN was constructed so that each CMO had a separate Propensity to Change node (S6 Table). This allowed each of the CMO interventions to be examined separately. A CPT was developed for each intervention. CPTs were of the form shown in Table 2. The parent nodes for each of these CPTs were Household, Knowledge, Culture and Trust. Further inputs for the interventions included prior uptake of targeted behaviour, customer segmentation and the impact of the intervention for the households in each of the High, Low and Nil Propensity to Change states.

**Table 2:** Example CPT to be completed.

	CMO—Off-peak tariffs											
Household	Using segment distributions for Strategy for Queensland											
Knowledge	High				Medium				Low			
Culture	High		Low		High		Low		High		Low	
Trust	High	Low	High	Low	High	Low	High	Low	High	Low	High	Low
High	0.95	0.70	0.80	0.60	0.80	0.60	0.70	0.50	0.60	0.20	0.10	0.00
Low	0.05	0.20	0.20	0.30	0.20	0.30	0.20	0.25	0.30	0.50	0.20	0.10
Nil	0.00	0.10	0.00	0.10	0.00	0.10	0.10	0.25	0.10	0.30	0.70	0.90

The change impacts used in the Appliance usage node were identified in the industry workshops using industry data and expertise to specify the percent or wattage reduction or increase in peak electricity demand by those households which were in a High, Low or Nil change state for each CMO. These values were used to calculate the change in peak demand for electricity after intervention implementation. The reduction in peak demand was calculated by the modelled percentage of the householders who are in a given state (High, Low or Nil) for each CMO determined from the BN and the demographic targeted. To take account of the change in demand by the non-targeted demographic cluster, 25% of the remaining households were combined with the Low state value. The reduction (or increase) in network peak demand and the uncertainty of the values were derived in the workshops with the industry partner experts using industry data [27]. The interaction of the percent in each state (reduced by the percent already in those states) and the reduction in network peak demand was calculated based on these key elements of the model.

The model was quantified for three localities: the whole state of Queensland, Townsville (a northern coastal town) and Toowoomba (a southern inland town).

#### Implementation

Since most technical data were provided by the working party members in the form of Microsoft Excel spreadsheets and in order to enhance the engagement with the BN model by the user group, the model was developed using spreadsheet software. Excel was chosen as this software is widely used and more easily understood and used by the intended users than is specialist BN software. The workbook was structured with separate sheets for each of the nodes of the system. The marginal probabilities for a given node were calculated by algebraically combining the probabilities of each of the parent nodes being in each state (eg High, Low or Nil) using the CPTs for that node [30]. The spreadsheet was structured to facilitate the checking of the validity of this implementation.

#### Communication

A key area of interest in this model was to engage stakeholders in the process from model development to model use. A key factor in communication is visualisation, which may be applied to enhance understanding of areas of interest, and the forms of visualisation may come in many forms [31]. Two of these methods, information visualisation, representing the system as a tree diagram, and data visualisation, using tables and graphs, were used to present the model to the industry participants.

**Fig 4:**
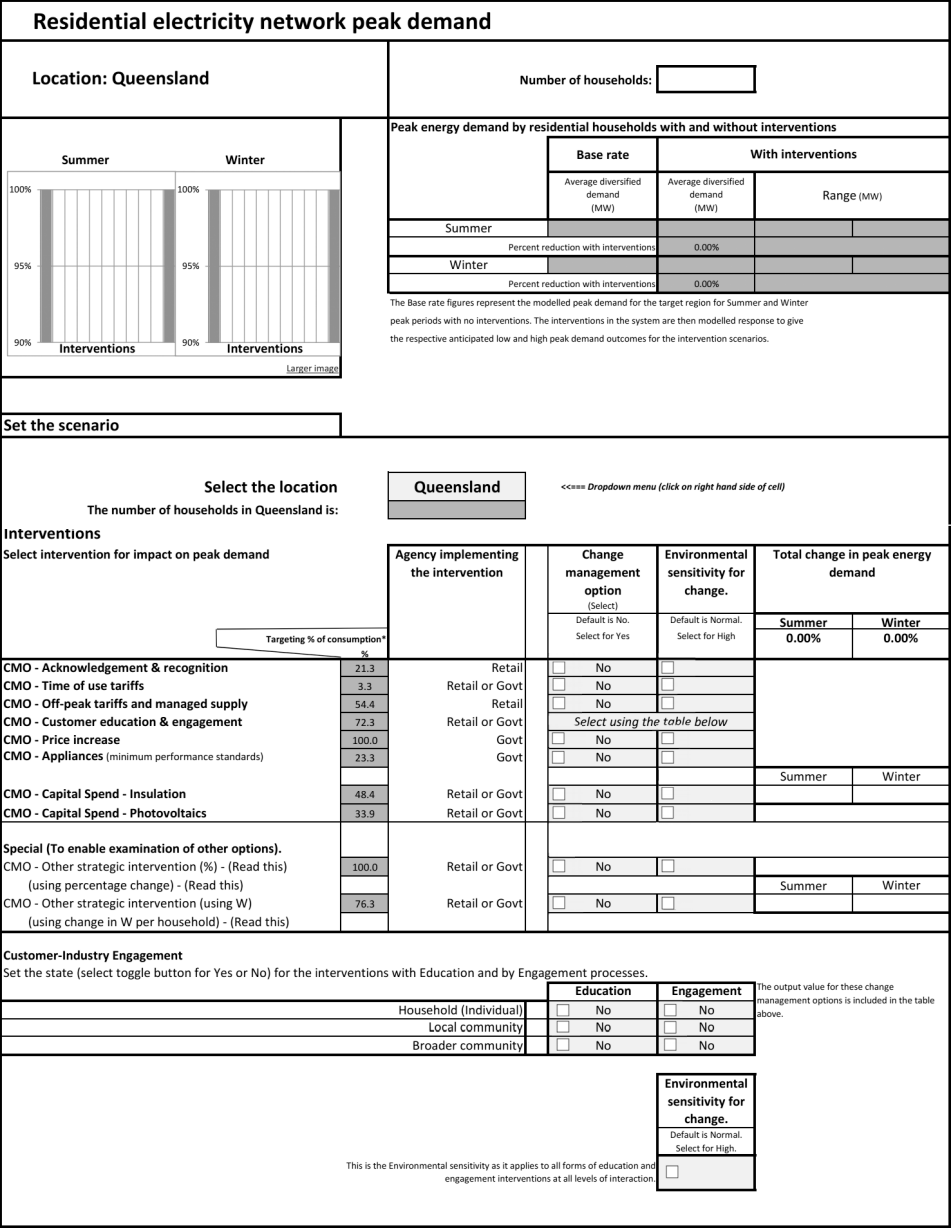
Dashboard for scenario selection and summary outputs.

A dashboard interface was added to the spreadsheet to allow users to interact with the BN through the simple process of selecting scenarios using tickboxes. The dashboard is depicted in Fig 4. The data entry needed was colour coded to indicate whether it referred to relatively stable items or ones requiring updating for a particular scenario.

The outputs were provided to users visually in waterfall charts in which the changes in peak demand are presented in a cumulative manner. The peak demand starts at 100% on the left hand side and changes with each CMO to the level on the right hand side (100% minus the cumulative percent reduction). This allows the individual contributions to the reduction in peak demand of each of the CMOs and their total impact to be clearly understood. For the sake of presenting the outputs, a scenario with all the options selected as being present was implemented.

To facilitate model validation (see below) and to enable a wider range of inferences, the BN component of the model was also constructed using GeNIe software [32], a dedicated software tool for building and calculating BNs. This software provides a range of tools to examine a model including a strength of influence tool that displays the relative influence a particular node has on its child nodes.

#### Validation

The validity of a model is reflected in its ability to describe the system being modelled through both its output and the mechanism by which that output is generated [33]. The sources of confidence in BN validity using the framework for validating a BN model that has been proposed by Pitchforth and Mengersen [33] are based on assessing the validity of the model structure, the states assigned to each node and the probabilities assigned to these states. The validation process thus applies to each model element and to the model as a whole.

The network peak demand model was validated through internal validation and validation by the working group and other industry partner workshops. Internal validation involves checking the probabilities in the BN for consistency. Sensitivity analysis, an approach described in the next section, also allows validation of the model through checking that the model behaves broadly as expected. Stakeholder validation involves critical review of the Bayesian network and model design and outputs by stakeholders.

Internal validation was undertaken by members of the research team. The consistency of the probabilistic inputs and outputs in the BN was confirmed through careful inspection of the coherence and impact of parent node CPTs on child node CPTs throughout the model. Stakeholder validation of the model functioning and outputs was undertaken through the research workshops and other meetings with industry partner and academic project member experts in the field. This provided stakeholders with the opportunity to inspect and use the model and to provide feedback.

The conceptual model was validated for internal consistency during the Design phase [26], with further validation during the workshops with industry partners as part of the development of the BN. This process was also used for defining and verifying the node states. To aid validation of the outputs of the model, as well as provide for interpretation later in the use of the model, key intermediate outputs were presented in the spreadsheet as graphical outputs, as described in the previous section. The Excel implementation was also validated by checking each CPT output against a partial model constructed, for the social components of the conceptual model, using GeNIe software.

#### Sensitivity analysis

A sensitivity analysis was conducted through an iterative process to both validate the model and to allow the project users to see the feedback from their own quantification of the variables. One-way analysis, whereby one variable is changed while the other variables are kept constant, is a widely known and applied sensitivity analysis approach [34]. The changes in the probabilities of nodes when changing another node should conform broadly as expected. The approach taken was the direct examination of various set states of the input nodes of the CPT, and by selecting scenario settings that provided findings in a BN sense. For example, the influence of selecting various combinations of Yes or No for Education activities and Engagement activities at the Broader community, Local and Householder levels were examined for their flow through impact on the Customer-Industry Engagement node to its child, Trust and Knowledge nodes. The outputs of the model, both intermediate and end point, were examined by the expert group and the working group who checked the validity of the outputs based on their internal social market research and their knowledge of the householder demand response. The impact on the states of the nodes of the system from changing the selected tickboxes on the dashboard were observable in graphics provided on relevant sheets of the spreadsheet model. Further examination of the model was conducted through investigating the impact on the states of the CMOs when all states of the input nodes are set to High then each of the input nodes is set to Low while the other two input nodes set to High. An additional examination of the Propensity to Change node to the states of its parent nodes was undertaken to test its sensitivity to different levels of Education and Engagement activities. With this, the evidence was set for each CMO either with the CMO only, the CMO with no associated Education or Engagement activities, the CMO with associated Education activities at the Broader and Local Community levels and the CMO with additional associated Education and Engagement activities at the Household level.

#### Evaluation

The BN model can be used to investigate the major influences in the system and to assess the impact of different combinations of interventions. Operationally, these interventions and sensitivity changes can be implemented in the BN spreadsheet and underlying CPTs.

Scenario assessment allows stakeholders to explore impacts under alternative or ‘what if’ conditions. The model was developed to allow different scenarios to be selected within the spreadsheet. After selecting a locality (eg Queensland, Townsville, Toowoomba) a combination of the change management options (Acknowledgment & Recognition, Time of Use Tariffs, Off-Peak Tariffs and Managed Supply, Customer Education & Engagement, Price Increase, Appliances (minimum performance standards), Capital Spend–Insulation, Capital Spend–Photovoltaics) could be selected. The spreadsheet model also had provision for examining other localities and other CMOs with the entry of appropriate input data. It also provided for including an Environmental sensitivity for change context (defined in S1 Table) for each option. This could be set to Normal or High to enable the examination of impact under different contexts of the social understanding of the need for reducing peak demand. The outputs with these latter implementations are not described in this paper.

## Results

The results from each scenario that was run with the model, in terms of the projected effect on network peak demand, were provided in chart and table form (see for example, Fig 4 and Table 3). The model also provided a graphical output of the intermediate states. The results indicate that the impact on network peak demand depends on the strength of influence combined with the proportion of the households targeted by a particular intervention and the impact on demand by those households being the High, Low or Nil state of propensity to change. These effects are described under the following headings: Main factors impacting on outcomes; Validation; Sensitivity analysis and Scenario testing.

**Table 3:** Change in peak demand for Queensland.

**Impact for each Change Management Option with different levels of Education and Engagement activities**			
**Change Management Option**	**CMO only**	**CMO with Education**	**CMO with Education & Engagement**
Acknowledgment & Recognition	-0.12%	-0.13%	-0.15%
Time of Use Tariffs	-0.05%	-0.11%	-0.16%
Off-Peak Tariffs and Managed Supply	-0.47%	-1.57%	-2.42%
Customer Education & Engagement		-0.42%	-0.76%
Price Increases	-1.68%	-2.64%	-3.38%
Appliances (minimum performance standards)	-0.23%	-0.23%	-0.23%
Capital Spend–Insulation Summer	-0.27%	-0.55%	-0.78%
Winter	-0.24%	-0.48%	-0.67%
A negative value indicates a reduction in network peak demand			
**CMO only:**	Change Management Option selected with no associated Education or Engagement activities		
**CMO with Education:**	Change Management Option selected with associated Education activities at the Broader and Local Community levels		
**CMO with Education & Engagement:**	Change Management Option selected with associated Education and Engagement activities at the Household level		

### Main factors impacting on outcomes

The main factors impacting outcomes were revealed by the sensitivity analysis and scenario testing. This was further emphasised by the examination of the relative strength of influence revealed by GeNIe. The relative strength of influence of the nodes in the BN is depicted in Fig 3. The thickness of the arrows in this figure depicts the relative strength of influence of the parent node on the child node. This analysis revealed differences in the impact of the Propensity to Change nodes for each of the CMOs. For some of the nodes, Culture has the stronger influence. This is evident for the Acknowledgment and Recognition option and also for the Propensity to Change with the Capital Spend–Insulation option. Knowledge had a greater influence in the Time of Use, Off-Peak Tariffs and Managed Supply and with Price Increases. Trust had a lesser strength of influence than the other parent nodes on the fulcrum nodes of Propensity to Change. However, Trust had a greater influence on the Time of Use, Off-Peak Tariffs and Managed Supply and with Price Increases nodes.

**Fig 5:**
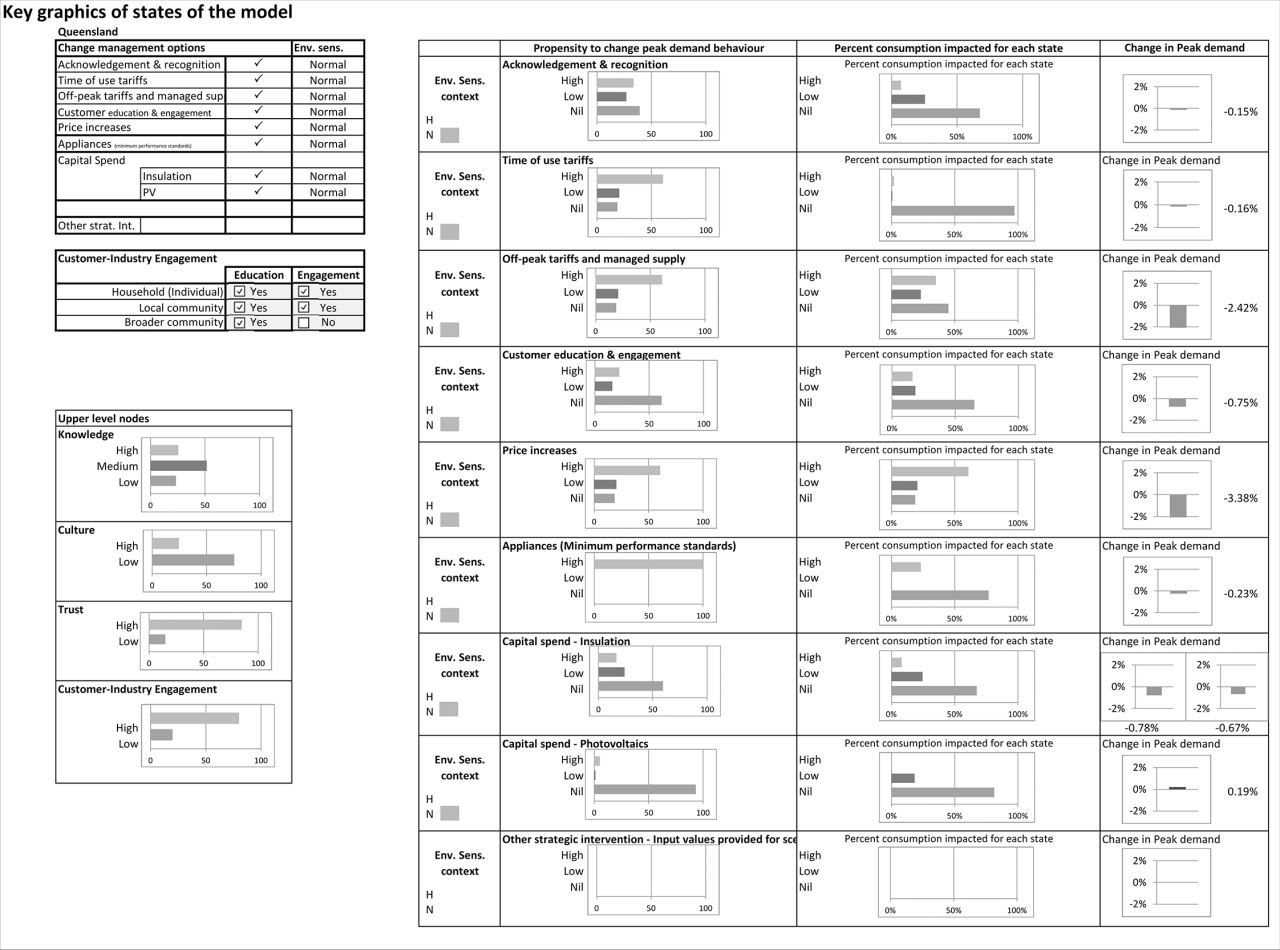
Intermediate outputs and impacts on peak demand.

The CMO selected and the states of the upper level input nodes, Knowledge, Culture, Trust and Customer-Industry Engagement is visualised in Fig 5. This figure presents for each CMO, the states of the householders’ Propensity to Change behaviour, the percent of the network peak demand consumption by the customer segments being impacted by the intervention, and finally on the right, the percent change in network peak demand for each intervention. The High and Medium states of the Knowledge node were increased with Education and then further with the combined Education and Engagement activities. With increased Education and Activity levels, there was a correlated increase in the High state of the Customer-Industry Engagement node. The Trust node was strongly related to Education and Engagement. This last relationship is discussed in more detail below in the ‘Sensitivity analysis’ section.

### Validation

The validation of the BN model aimed at checking the Excel implementation, the states of the nodes of the model and the outputs produced (Fig 5), and the behaviour of the model in the sensitivity analysis, described below. The output review process assessed that the model was producing outputs within expected ranges for the Upper Level Nodes of the model, the Propensity to Change nodes, the Percent Consumption Impacted by Each State and the Change in Peak Demand charts.

### Sensitivity analysis

**Fig 6:**
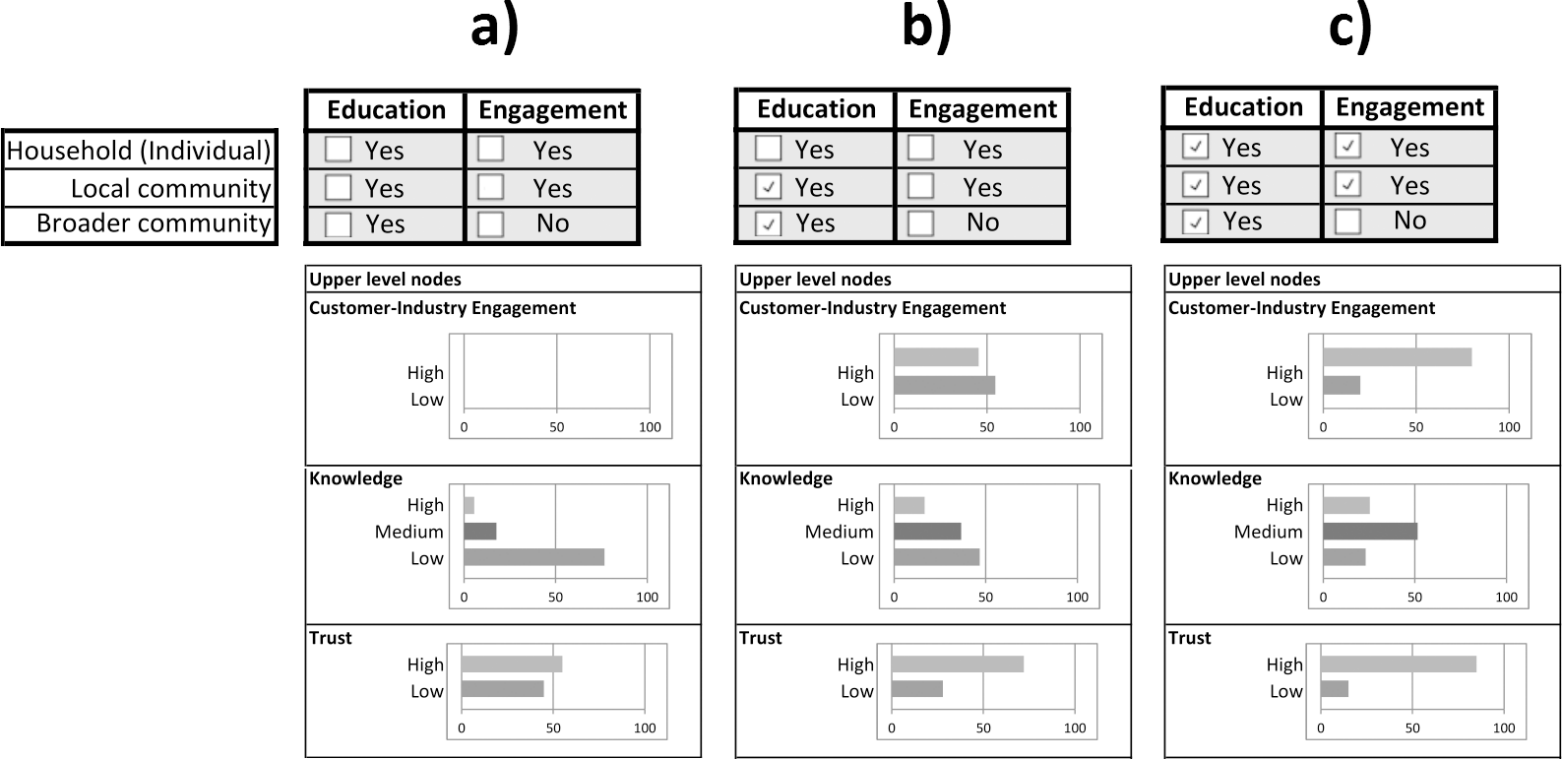
Sensitivity analysis with different levels of customer education and engagement activities.

A sensitivity analysis was conducted by investigating the impact of inputs on key nodes of the model. Fig 6 shows the sensitivity of the Customer-Industry Engagement, the Knowledge and the Trust nodes to changes in the evidence of the states of the Education and Engagement sub-network nodes. Changing the selection from no Education and Engagement activities, Fig 6 (A), through to a higher level of Education and Engagement activities, Fig 6 (C), increased the probability of a High level of Trust by over 50% (from 0.55 to 0.85). The probability of a High level of Knowledge increased more than four-fold (from 0.06 to 0.26). Similarly, the probability of High Customer-Industry Engagement changed from nil when there were no activities to 0.46 with moderate Education levels and 0.80 when the activities for both Education and Engagement were at a High level.

The analysis of the sensitivity of the states of the CMO Propensity to change nodes (S5 Table) showed that the effect on the levels of the states varied differently across the CMOs with inputs of parent nodes. The impact of changing each of the parent nodes to a Low state, with the others being maintained as High, led to different levels of reductions for each of the CMOs. When Culture was Low, the probability of a High Acknowledgment & Recognition option reduced from 0.95 to 0.3, compared to reductions of 0.7 when the other two parent nodes were each changed to a Low state. Trust had the greatest impact on Off-Peak Tariffs and Managed Supply. The probability of a High state for this CMO reduced from 0.95 to 0.6 when the level was Low. This CMO was also sensitive to the level of Knowledge.

A CMO which was sensitive to changes in social influences was Capital spend–Insulation. With this CMO, the probability of a High level of Spend collapsed from 0.65 when Culture, Knowledge and Trust were High, to 0.01, 0.05 and 0.00 when each of these influences was set from High to Low. The S6 and S7 Tables show in further detail the impact on peak demand for the households in a High or Low state of Propensity to Change, and the corresponding uncertainty of these values.

### Scenario testing

**Fig 7:**
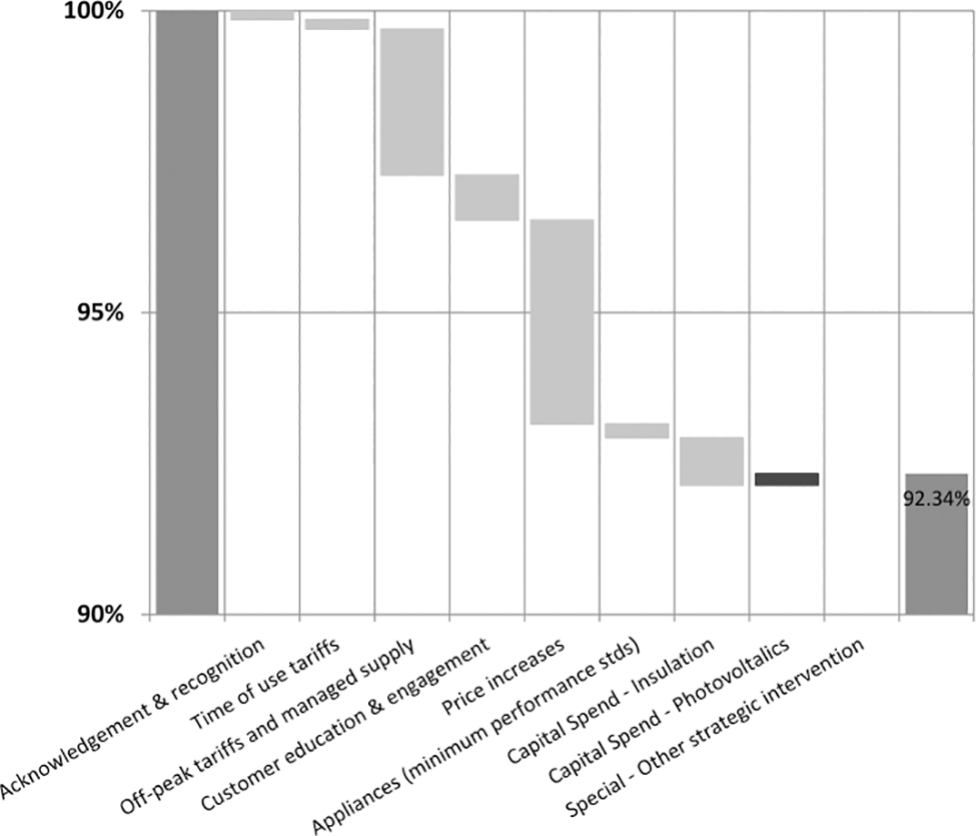
Waterfall chart of change in peak demand–Queensland–Summer.

The ‘what if’ options of scenario testing revealed the impact of combining various levels of Education and Engagement activities with each of the CMOs and the broad differences between the three localities, namely: Queensland, Townsville and Toowoomba. Table 3 shows the impact for Queensland for each of the CMOs with different associated Customer-Industry Engagement activity levels. Table 4 presents the changes in peak energy use across the three localities and the impacts of the different CMOs. An example of how these impacts are presented in the spreadsheet in graphical form is shown in Fig 7.

**Table 4:** Change in peak energy demand with interventions–Queensland, Townsville and Toowoomba.

Change Management Options	Queensland		Townsville		Toowoomba	
	Summer	Winter	Summer	Winter	Summer	Winter
Total change	-7.85%	-7.74%	-8.08%	-6.82%	-7.55%	-8.10%
Acknowledgment & Recognition	-0.15%		-0.13%		-0.17%	
Time of Use Tariffs	-0.16%		-0.14%		-0.13%	
Off-Peak Tariffs and Managed Supply	-2.42%		-2.12%		-2.60%	
Customer Education & Engagement	-0.76%		-0.76%		-0.75%	
Price Increase	-3.38%		-3.38%		-3.38%	
Appliances (minimum performance standards)	-0.23%		-0.24%		-0.20%	
	Summer	Winter	Summer	Winter	Summer	Winter
Capital Spend–Insulation	-0.78%	-0.67%	-1.52%	-0.25%	-0.52%	-1.08%
Capital Spend–Photovoltaics	0.19%		0.19%		0.19%	

This scenario provides the outputs with the Customer-Industry Engagement options of Broader Community Education activities checked and both Education and Engagement activities for Local Community and Households checked.

Fig 7 shows the waterfall chart for Queensland for summer with interventions and a High level of associated Customer-Industry Engagement processes. The biggest reductions in peak demand were with Price Increase, then the Off-Peak Tariff and Managed Supply intervention, followed by Customer Education and Engagement and Insulation. There is a negative effect for photovoltaics, where their introduction increased peak demand. This phenomenon is discussed further below.

## Discussion

The network peak demand model provides a decision support tool and a means of understanding the key influences and impacts on network peak demand. The model allowed various intervention scenarios to be tested which provided insights into how the system could be managed.

The use of a BN enabled the combination of information from diverse sources. Fundamental to the modelling approach was an integration of aspects of the housing type, the appliances in them and the broad physical environment with the propensity of households to alter their consumption behaviour based on a number of characteristics. Additionally, the model was able to take account of the social aspects of the solution-finding participants of the energy retailer, government and households. The model was constructed at a level of complexity and detail commensurate with available data and expertise.

The sensitivity analysis that was carried out in the development process showed that work should be focussed on ensuring that the accuracy of the parameters for Trust, Culture and Knowledge are improved. Further, the large impact of the sub-elements of the Education and Engagement sub-nodes on the intermediate nodes and on the Propensity to Change nodes indicates the importance of defining the activities that are driving these influences. These system elements are key drivers and need to be included as part of the design of interventions to reduce network peak demand. The analysis of the impact of the parent nodes on the fulcrum nodes of the Propensity to Change for each CMO is, in fact, a re-expression of the probability distribution that was established for the nodes. However, the examination of the impact highlighted to the industry participants, the key areas where further customer research would yield the greatest benefit toward strengthening the model, and of understanding drivers of peak demand behaviours and appropriate interventions.

Applying interventions changed the states of key intermediate nodes and these changes resulted in reducing network peak demand. For the modelled intervention conditions, it was found that increasing Education and Engagement could increase the level of the High state of Trust by 50% and Knowledge by a factor of six. The increase of the High states of these intermediate nodes was seen to flow through to a reduction in network peak demand. It is therefore important that intervention strategies take into account the impact on Trust and Knowledge of activities and programs that are implemented.

A significant finding of this study is that the application of the model, through the use of CMO interventions, provides insight for policy. The modelled CMOs generally reduced peak demand. However, maximum impact was achieved when well-designed interventions included effective engagement with residential customers. For example, price signals and tariffs and managed supply combined with customer engagement activities aimed at influencing change, produced greater reductions in peak demand. It was observed that network peak demand was reduced by nearly 3.4% for the Price Increase option when combined with a High level of Education and Engagement activities. A reduction of 2.4% was seen for Off-Peak Tariffs and Managed Supply. These reductions in peak demand were five and two times greater, respectively, than for those interventions without any Education and Engagement activities. On the other hand, the introduction of government CO_2_ reduction initiatives, focusing on increasing the uptake of photovoltaics (PVs), led to a perverse effect of an increase in peak demand. The customers with PV systems were able to obtain a price advantage by shifting their energy use to the evenings (peak time). The challenge, therefore, is to ensure that interventions are well designed through implementing systems-driven, integrated approaches incorporating customer engagement initiatives.

### Strengths of the approach

There are three major strengths of this BN approach to understanding customer energy use, namely stakeholder engagement, multiple interactions and quantified output. The socio-technical system of network peak demand has at its core the householders who either implement capital expenditure on peak energy saving changes, install devices that can alter peak demand behaviour of appliances, or alter their behaviour to use electricity for some activities outside the peak demand periods. The stakeholder engagement and elicitation process makes explicit the meanings of key elements and information needs. Involving key stakeholders in the model development and parameterisation facilitates shared understandings and meanings for elements that are traditionally difficult to measure. Using expert elicitation for unavailable data provides a method for using qualitative data to compute outcomes. By constraining the elicitation to specific, limited causal relationships, BNs overcome the cognitive difficulties people have with applying heuristics when making decisions [35]. The scope of data being elicited allows stakeholders to focus on relationships that they can more easily determine.

Secondly, the process of developing measures for the model also identifies specific informational needs and gaps that exist in industry data sources. This approach enables targeted industry data collection and the meaningful use of the data within an industry relevant model. A variety of data sources were used in this modelling such as household numbers, modelling of heat loads in houses with differing insulation, expert elicited CPTs and industry survey data. A strength of modelling using BNs is that it is able to accommodate this diversity.

A BN model built on expert predictions and reliable data is a powerful tool for understanding the complexity of current energy use and predicting future trends. BNs are an approach that can also be used to test future scenarios, and key stakeholders can use this model in organisational planning to explore complex interactions and develop shared understandings.

Thirdly, the BN modelling approach enables users to combine multiple interactions and produces a quantified output. The model allows for synergies between interventions, and for others to be made explicit, for example, the Customer education and engagement option. The constituent CPTs can be adapted as new information is provided and the process of quantifying the BN can highlight knowledge and information gaps.

The model provided a measure of reduction in Network peak demand from a number of interventions. It indicated that with a combination of well-designed interventions, a reduction of 5–7% could be achieved. In the context of the state of Queensland, this would represent a reduced peak demand of approximately 150 megawatts (MW) across the network [3] helping to avoid or delay network upgrades that would otherwise be required. This modelled reduction was considered by some industry experts to reflect a likely diversified energy impact for Queensland as a whole and is comparable with the 8.1% market potential of demand response by residential customers estimated by Faruqui and colleagues [6]. However, the reductions were seen by some as being potentially conservative. A recent trial using tariff measures across three regions in Queensland led to a 19% reduction in peak electricity demand by the participants on specified ‘event days’ [36]. This indicates that with a small network that was reaching capacity, the development of targeted Customer-Industry Engagement activities with appropriate CMOs, larger reductions than those modelled could be obtained. On the other hand, these reductions may be an overestimate as a difficulty in choosing the ‘event days’ for this trial resulted in the weather on some of those days being milder than predicted. Thus, the reduction observed may not have occurred if, for example, comfort levels would have been impacted by people not turning on their air-conditioning on the designated extreme days.

In the study under consideration here, the impact values obtained for the proportion of households in the High state (S6 Table) did not exceed 10% (0.1) reduction in demand, suggesting that this is an area for refinement. However, irrespective of the actual figure for the likely reduction in peak demand, the model highlights that it can provide estimates of reduction in peak demand and further emphasises the importance of designing interventions which target the key drivers in the system and do not create barriers to adoption or perverse outcomes, as in the case of incentives for uptake of PVs. By considering these findings, planning appropriate interventions can lead to significant savings and prevent the rises in electricity charges that are influenced by this network cost.

Finally, the BN model highlights the interactions of the feed-in nodes. For example, introducing an intervention such as Off-Peak Tariffs and Managed Supply without the concurrent application of an Education and Engagement strategy produces a smaller change in peak demand than is predicted with such a strategy in place. Revealing and making explicit such synergies is a strength of systems thinking in a BN.

### Limitations of the approach

While BNs are powerful tools that can produce useful results, they do require assumptions and approximations. The structure of the conceptual model and the assumptions need to be as clear as possible within the context of its scope.

The model was restricted to key elements and interactions because more nodes and states of those nodes raise the complexity of the problem exponentially and it can become more difficult to quantify the additional parameters and nodes in an over-complicated model. Although the model included sub-node interactions, the interactions between the CMOs’ sub-nodes were restricted to the Customer Education and Engagement option with the other options and not between those other options. The model was built with the same influence of the Trust and Knowledge nodes for each of the CMOs, which is a potential problem. This could be enhanced to reflect the different types of trust and knowledge that would exist for interventions with differing complexities of trust and understanding [37]. With the Trust node, for example, it is recognised that programs that are developed with the aim of reducing network peak demand may have differing levels of trust required to impact on this demand.

While model complexity is an issue, a key element that has been identified is Trust. Future versions of this model could categorise the different interventions into those where the perceived consequences of Trust regarding an intervention is either low, intermediate or high. A low perceived consequence for Trust would arise where the householder is able to independently ascertain information. A high perceived consequence of Trust in public institutions or energy providers could be in situations where Trust is important such as allowing the external control of services (eg cooling) or with privacy issues to do with the installation of ‘Smart Meters’. With the Knowledge node, the prior knowledge and the ease of acquiring the required knowledge and skills for different interventions could be similarly categorised to take account of requirements of different interventions. Modelling these additional interactions would, however, not only substantially increase the complexity of the model but also the information required to be obtained from industry experts, or other sources.

The quantification of the BN model can combine data that reflects the knowledge and expertise of the key stakeholders. This is both a weakness and a strength. If the experts have limited knowledge, they will need to seek additional expert data or develop ways of obtaining the required data. The BN allows for the testing of these different data sources to explore impact or relevance.

The model implementation work revealed several gaps in the information available from surveys or collected data sources. For example, the model provides a post-intervention proportion of the target households which will be at a High or Low state of Propensity to Change. As the model is interested in changes, the proportion of the target households which may have already been in those states is used to provide a measure of the change of states and that flows through to a resultant reduction in network peak demand. An estimated value for the prior state of the target population was used to provide an assessment of the change of proportion of the target households. As this is an important component of the model, it highlighted a further area for the energy utilities to focus their market research.

### Analysing further change management option impacts

It is important to note that the model assumes that any change management programs that are instigated will be based on household enablement and participation in solution-finding towards achieving desired outcomes. The model thus relies on any interventions being designed and undertaken with the aim of achieving the desired outcomes. Without this design, including participative solution-finding, it is unlikely that the desired level of reduction of network peak demand will eventuate, which is both a strength and a limitation. The forced consideration of well-designed programs within a systems context should lead to better outcomes for a particular level of investment.

## Conclusion and Implications

The model of socio-technical elements of the network peak demand system was built on the basis that an integrated approach is required that combines both the social and the technical. Previous approaches that apply either sociological or technical solutions to overcome network peak demand problems have not been successful in developing a quantifying model. The mathematical modelling using a systems approach, with a BN implementation, enabled the quantification and combining of information from diverse sources.

The model showed that network peak demand from an intervention combined with a High level of Customer-Industry Engagement activities could be reduced by over 3% using a single intervention and up to 8% for a combination of CMOs. As improvements are made to the model, more precise outputs will be obtained.

The interactive nature of the model development and its use provide benefits to users. Stakeholders engaging with the quantification process and with using this model will be able to use the insights that can be obtained from it to achieve improved management strategies in the electricity system. The modelling process highlights gaps in our knowledge and where further information needs to be collected. As this information is gathered, iterative improvements can be made with the model and to the values, resulting in more reliable estimates and understanding of which combination of interventions to use in particular locations. This will help to design programs that utilise current understanding of the interlinked sociological perspectives of well-designed interventions that deliver improved network utilisation and reducing infrastructure costs that would otherwise need to be built to meet increasing peak demand.

The participative approach to developing the model promotes shared understanding of the key elements of the system and their interactions and also aids the identification of the key information gaps that could be filled to further strengthen the model. The work reported in this paper also highlights the potential of this modelling approach for investigating other complex socio-technical domains and providing similar insights that may guide their management.

## Supporting Information

S1 PDF Data underpinning the quantification of the conceptual model. Data collected, developed and used to quantify the conceptual systems model to be implemented through a Bayesian network. The data collected in the workshops and used to develop the BN consists of: Number of households in region or location, Level of Prior Knowledge, Trust and Culture, the Prior states for uptake of behaviours being targeted, the reduction in Energy demand impacted by heat load with insulation. The CPTs for the modelling are detailed for Customer-Industry Engagement (CIE), Knowledge, Trust and Culture. The Electricity consumption by Region or locality which is combined in the model with the CPT for Propensity to Change and Strategic Action Clusters for each of the Change Management Options (CMOs), and the Diversified energy demand by household appliances for Queensland and other localities.
(PDF)

S2 PDF A description of the model. The conceptual model, Fig 1 in the paper, was developed as the first phase of a larger project and reported in more detail in Buys and colleagues [26]. The figure presents the major components of the system. However, each component consists of sub-networks. In the quantified system, these components produced outputs based on the states of the nodes and the sub-networks that each component contained. There are three broad groupings or domains of the conceptual model components–social, technical and stakeholders–and the sub-networks are described in the following sections along with a description of how the domains are brought together. Further description and definitions of the nodes and other items are presented in Supporting Information (S1, S2, S3 and S4 Tables).
(PDF)

S1 Table Nodes with probabilistic links in the Bayesian network. Description and definitions of the Nodes of the Bayesian network with a specification of the states of the nodes. These nodes are the Customer-Industry engagement, Knowledge, Trust, Culture, Environmental sensitivity–Context, and Propensity to Change.
(PDF)

S2 Table Nodes of the network which specify characteristics impacting on the model. Description of the elements of the system which impact the technical aspects of the model. These are the Physical environment, House, Appliances, Appliance usage (Household) nodes and their states.
(PDF)

S3 Table Description of nodes that are modelled by their influence on Change Management Options and interventions. Description of the Retail market strategies and the Government policy elements of the system.
(PDF)

S4 Table Change Management Options through retail market, government policy and customer-industry engagement. Description of the Change Management Options: Acknowledgment & Recognition; Time of Use Tariffs; Off-Peak Tariffs and Managed Supply; Customer Education & Engagement; Price; Appliances (minimum performance standards); Capital Spend—Insulation, Capital Spend—Photovoltaics and Other Strategic Interventions.
(PDF)

S5 Table Propensity to Change peak demand behaviour with changes in parent nodes. Table with values of outputs of the model for the Change Management Option nodes for the CIE inputs having Culture, Trust and Knowledge all High or with Culture Low*, or with Trust Low*, or with Knowledge Low*.
(PDF)

S6 Table Impact on electricity demand of households being in a High, Low or Nil state of Propensity to Change. Output from model for the Impact on electricity demand of households when they were in a High, Low or Nil state of Propensity to Change. Proportion or wattage impact on peak demand that will be reduced for each state. The data indicates, for example, 0.10 is a 10% reduction in peak demand and 280 watt reduction is from a reduction in heat load.
(PDF)

S7 Table Uncertainty applied to impact on peak demand for High and Low states of Propensity to Change. Table with values used in the modelling. The values are the uncertainty applied to impact on peak demand for High and Low states of Propensity to Change. Improvements in these values could be obtained for future iterations of the model development.
(PDF)
