# Supplementary material for: Systems Modelling of the Socio-Technical Aspects of Residential Electricity Use and Network Peak Demand
Source: PLoS One. 2015 Jul 30;10(7):e0134086. doi: 10.1371/journal.pone.0134086 (PMC4520613; doi:10.1371/journal.pone.0134086)
Supplement: S1 PDF — Data collected, developed and used to quantify the conceptual systems model to be implemented through a Bayesian network. The data collected in the workshops and used to develop the BN consists of: Number of households in region or location, Level of Prior Knowledge, Trust and Culture, the Prior states for uptake of behaviours being targeted, the reduction in Energy demand impacted by heat load with insulation. The CPTs for the modelling are detailed for Customer-Industry Engagement (CIE), Knowledge, Trust and Culture. The Electricity consumption by Region or locality which is combined in the model with the CPT for Propensity to Change and Strategic Action Clusters for each of the Change Management Options (CMOs), and the Diversified energy demand by household appliances for Queensland and other localities. (PDF) [file pone.0134086.s001.pdf]

Data collected, developed and used to quantify the conceptual systems model to be implemented through a Bayesian network

Physical environment Households in region or location

For shift in average electricity consumption, **+ve is greater than** that for **Queensland** &
-ve is less than that for **Queensland.**

Queensland	548,000	1*		Data from Ergon (2012) report
Townsville	40,000	2*	13.0%	
Toowoomba	70,000	2*	-11.0%	

1* - Ergon supplied data

2* - Based on population and household size data 2011

Prior Knowledge

	High	Medium	Low
Prior Knowledge (from	0.05	0.10	0.85

Trust

**Survey results**	Capital spend	
	High	Low
Trust in public institutions	0.65	0.35
Trust in energy providers	0.70	0.30

Culture

	High	Low
Public support for peak reduction	0.1	0.9
Public support for renewable sources of energy	0.8	0.2
Public support for mandated standards	0.5	0.5

Household

The information for Household (residential segment profile and consumption characteristics for the current scenario)

TABLE: Set values for prior states for uptake of behaviours being promoted by each of the Change management option interventions

Percentage of households' consumption already in each state

	Acknowledgement & recognition	Time of use tariffs managed supply	Off-peak tariffs and & recognition	Price increases	Appliances (Min. performance standards)	Capital spend - Insulation.	Capital spend - Photovoltaics	Customer education & engagement
High	5.0%	0.5%	15.0%	1.0%	0.0%	2.0%	0.1%	0.0%
Low	5.0%	0.5%	15.0%	1.0%	1.0%	10.0%	1.0%	0.0%
Nil	90.0%	99.0%	70.0%	98.0%	99.0%	88.0%	98.9%	100.0%

House (Energy demand impacted by heat load)

Reduction of peak energy demand from the Base rates for a household (watts per household)

**Cooling**	**Queensland**	**Toowoomba**	**Townsville**
From modelling	(with av. thermostat)	(with av. thermostat)	(with av. thermostat)
**Insulation**	watts	watts	watts
Nil change	0	0	0
Low change - Shift of 1 star rating*	180	100	400
High change - Shift of 2 star ratings*	280	200	600

*A shift of 1 or 2 star

**Heating**	**Queensland**	**Toowoomba**	**Townsville**
From modelling	(with av. thermostat)	(with av. thermostat)	(with av. thermostat)
**Insulation**	watts	watts	watts
Nil change	0	0	0
Low change - Shift of 1 star rating*	140	200	50
High change - Shift of 2 star ratings*	190	300	100

*A shift of 1 or 2 star

Customer-Industry Engagement (CIE)

Education	High		Low	
Engagement	High	Low	High	Low
Low	0.2	0.35	0.25	1
High	0.8	0.65	0.75	0
	1.00	1.00	1.00	1.00

Education

Household (Individual)	Yes				No			
Local community	Yes		No		Yes		No	
Broader community	Yes	No	Yes	No	Yes	No	Yes	No
Low	0	0	0.1	0.15	0.3	0.3	0.7	1
High	1	1	0.9	0.85	0.7	0.7	0.3	0
	1.00	1.00	1.00	1.00	1.00	1.00	1.00	1.00

Engagement

Household (Individual)	Yes				No			
Local community	Yes		No		Yes		No	
Broader community	Yes	No	Yes	No	Yes	No	Yes	No
Low	0	0	0.05	0.1	0.3	0.3	0.7	1
High	1	1	0.95	0.9	0.7	0.7	0.3	0
	1.00	1.00	1.00	1.00	1.00	1.00	1.00	1.00

Prior Knowledge

	High	Medium	Low	
Prior Knowledge	0.05	0.10	0.85	1.00

	High	Low	
Customer-Industry	0.46	0.55	1.00
engagement Trust	0.72	0.28	1.00

Prior knowledge	High				Medium				Low			
Customer-Industry	High		Low		High		Low		High		Low	
engagement Trust	High	Low	High	Low	High	Low	High	Low	High	Low	High	Low
Low	0	0	0	0	0	0	0	0.05	0.1	0.2	0.9	0.9
Medium	0	0.05	0.05	0.05	0.1	0.15	0.85	0.95	0.7	0.65	0.1	0.1
High	1	0.95	0.95	0.95	0.9	0.85	0.15	0	0.2	0.15	0	0

Trust

Trust in public institutions	High				Low			
Trust in energy providers	High		Low		High		Low	
Customer-industry engagement	High	Low	High	Low	High	Low	High	Low
Low	0.00	0.20	0.10	0.60	0.10	0.60	0.30	0.90
High	1.00	0.80	0.90	0.40	0.90	0.40	0.70	0.10

Culture

Public support for peak reduction	High				Low			
Public support for renewable sources of energy	High		Low		High		Low	
Public support for mandated standards	High	Low	High	Low	High	Low	High	Low
Low	0.1	0.2	0.15	0.3	0.7	0.9	0.9	0.9
High	0.9	0.8	0.85	0.7	0.3	0.1	0.1	0.1

Queensland Climate Zone

Queensland data Ergon Energy (2012)

Segments ordered per Ergon Energy (2012) report
(The % of total customer base, Average consumption and totals for each of the Consumption Bands is provided for interest. The information is not used in the model.)

Segment Consumption Band	Residential Segment profile	% of customer base	% of total consumption	Average consumption (per customer) (kWh pa)		
**High**	'Cash & Careers'	3.8%	5.1%	9933.7	16.4%	20.6%
	'Transition Blues'	3.6%	4.1%	8575.2		
	'Gen X Parents'	4.1%	5.3%	9588.6		
	'Flush Families'	4.9%	6.2%	9537.2		
**Medium**	'Beginnings'	12.2%	12.3%	7586.5	56.3%	58.1%
	'Taking Hold'	6.6%	7.1%	8065.8		
	'Gen X Singles'	6.8%	7.5%	8185.9		
	'Boomer Barons'	3.9%	4.3%	8177.5		
	'Modest Means'	22.0%	21.9%	7473.9		
	'Mature Wealth'	4.8%	5.0%	7741.8		
**Low**	'Golden Years'	3.8%	3.3%	6517.2	27.3%	21.3%
	'Active Elders'	10.9%	8.3%	5674.0		
	'Leisure Buffs'	9.5%	6.9%	5434.4		
	'Our Turn'	3.0%	2.8%	7046.2		
			100.0%	Calculated from data from Ergon Energy, 2012		

Toowoomba Climate Zone

From South West data Ergon Energy (2012)

Segment Consumption Band	Residential Segment profile		% of total consumption			
**High**	'Cash & Careers'		4.0%			19.3%
	'Transition Blues'		5.1%			
	'Gen X Parents'		5.0%			
	'Flush Families'		5.2%			
**Medium**	'Beginnings'		14.0%			56.0%
	'Taking Hold'		5.7%			
	'Gen X Singles'		4.9%			
	'Boomer Barons'		3.2%			
	'Modest Means'		23.8%			
	'Mature Wealth'		4.3%			
**Low**	'Golden Years'		2.9%			24.7%
	'Active Elders'		8.7%			
	'Leisure Buffs'		10.2%			
	'Our Turn'		2.9%			
Axiom report (2012)			100.0%			

Townsville Climate Zone

From Northern data Ergon Energy (2012)

Segment Consumption Band	Residential Segment profile		% of total consumption			
**High**	'Cash & Careers'		5.5%			21.9%
	'Transition Blues'		3.5%			
	'Gen X Parents'		6.5%			
	'Flush Families'		6.4%			
**Medium**	'Beginnings'		13.5%			59.5%
	'Taking Hold'		8.5%			
	'Gen X Singles'		9.0%			
	'Boomer Barons'		3.8%			
	'Modest Means'		19.7%			
	'Mature Wealth'		5.0%			
**Low**	'Golden Years'		3.0%			18.6%
	'Active Elders'		7.3%			
	'Leisure Buffs'		5.2%			
	'Our Turn'		3.1%			
			100.0%			

Propensity to Change

Definition: A natural or acquired tendency, inclination, or habit in a person to make desired change of either capital spend or ongoing behaviour change.

Questions. To aid in the updating of the Conditional probability tables (CPTs), a question is posed for each one.

The impact of these interventions is captured in the 'Appliance usage' node where they are applied to the specified Customer Segments targeted.

This CMO is : **Inactive**

Question for completing the CPT: For each combination of the states of the input nodes, Knowledge, Culture and Trust (eg Medium, High, High states), what is the probability that the state of the Acknowledgement & recognition change management option with respect to modifiying peak demand be in a High, Low or Nil state? (This question is to be answered remembering that it is not asking the probability of the likely impact of this item given the states of the imput nodes; the impact of this item is addressed with an impact measure in a later sheet.)

Change Management Option	**CMO - Acknowledgement & recognition**											
Household	Using segment distributions for Strategy for Queensland											
Knowledge	High				Medium				Low			
Culture	High		Low		High		Low		High		Low	
Trust	High	Low	High	Low	High	Low	High	Low	High	Low	High	Low
High	0.90	0.70	0.30	0.10	0.70	0.60	0.20	0.10	0.70	0.70	0.20	0.10
Low	0.10	0.20	0.50	0.30	0.20	0.20	0.30	0.10	0.20	0.20	0.20	0.10
Nil	0.00	0.10	0.20	0.60	0.10	0.20	0.50	0.80	0.10	0.10	0.60	0.80
	1.00	1.00	1.00	1.00	1.00	1.00	1.00	1.00	1.00	1.00	1.00	1.00

This CMO is : **Inactive**

Question for completing the CPT: For each combination of the states of the input nodes, Knowledge, Culture and Trust (eg Medium, High, High states), what is the probability that the state of the Time of use tariffs change management option with respect to modifying peak demand be in a High, Low or Nil state? (This question is to be answered remembering that it is not asking the probability of the likely impact of this item given the states of the imput nodes; the impact of this item is addressed with an impact measure in a later sheet.)

Change Management Option	**CMO - Time of use tariffs**											
Household	Using segment distributions for Strategy for Queensland											
Knowledge	High				Medium				Low			
Culture	High		Low		High		Low		High		Low	
Trust	High	Low	High	Low	High	Low	High	Low	High	Low	High	Low
High	0.95	0.70	0.80	0.60	0.80	0.60	0.70	0.50	0.60	0.20	0.10	0.00
Low	0.05	0.20	0.20	0.30	0.20	0.30	0.20	0.25	0.30	0.50	0.20	0.10
Nil	0.00	0.10	0.00	0.10	0.00	0.10	0.10	0.25	0.10	0.30	0.70	0.90
	1.00	1.00	1.00	1.00	1.00	1.00	1.00	1.00	1.00	1.00	1.00	1.00

This CMO is : **Active**

Question for completing the CPT: For each combination of the states of the input nodes, Knowledge, Culture and Trust (eg Medium, High, High states), what is the probability that the state of the Off-peak tariffs change management option with respect to modifying peak demand be in a High, Low or Nil state? (This question is to be answered remembering that it is not asking the probability of the likely impact of this item given the states of the imput nodes; the impact of this item is addressed with an impact measure in a later sheet.)

Change Management Option	**CMO - Off-peak tariffs**											
Household	Using segment distributions for Strategy for Queensland											
Knowledge	High				Medium				Low			
Culture	High		Low		High		Low		High		Low	
Trust	High	Low	High	Low	High	Low	High	Low	High	Low	High	Low
High	0.95	0.70	0.80	0.60	0.80	0.60	0.70	0.50	0.60	0.20	0.10	0.00
Low	0.05	0.20	0.20	0.30	0.20	0.30	0.20	0.25	0.30	0.50	0.20	0.10
Nil	0.00	0.10	0.00	0.10	0.00	0.10	0.10	0.25	0.10	0.30	0.70	0.90
	1.00	1.00	1.00	1.00	1.00	1.00	1.00	1.00	1.00	1.00	1.00	1.00

This CMO is : **Active**

Question: For each combination of Knowledge, Culture and Trust (eg Medium, High, High) state of the input nodes, what is the probability that the state of 'Customer

Customer education & engagement

Change Management Option	**CMO - Customer education & engagement with the level of engagement being in State = High**											
Household	Using segment distributions for Strategy for Queensland											
Knowledge	High				Medium				Low			
Culture	High		Low		High		Low		High		Low	
Trust	High	Low	High	Low	High	Low	High	Low	High	Low	High	Low
High	0.80	0.60	0.10	0.10	0.80	0.50	0.20	0.10	0.60	0.20	0.10	0.00
Low	0.10	0.20	0.20	0.20	0.10	0.30	0.20	0.20	0.30	0.50	0.20	0.10
Nil	0.10	0.20	0.70	0.70	0.10	0.20	0.60	0.70	0.10	0.30	0.70	0.90
	1.00	1.00	1.00	1.00	1.00	1.00	1.00	1.00	1.00	1.00	1.00	1.00

Change Management Option	CMO - Customer education & engagement with the level of engagement being in State = Low											
Household	Using segment distributions for Strategy for Queensland											
Knowledge	High				Medium				Low			
Culture	High		Low		High		Low		High		Low	
Trust	High	Low	High	Low	High	Low	High	Low	High	Low	High	Low
High	0.10	0.10	0.00	0.00	0.00	0.00	0.00	0.00	0.00	0.00	0.00	0.00
Low	0.20	0.20	0.10	0.10	0.10	0.10	0.00	0.00	0.00	0.00	0.00	0.00
Nil	0.70	0.70	0.90	0.90	0.90	0.90	1.00	1.00	1.00	1.00	1.00	1.00
	1.00	1.00	1.00	1.00	1.00	1.00	1.00	1.00	1.00	1.00	1.00	1.00

Where the **High** state of Customer education & engagement of **0.455** & **Low** state of **0.545** are use for the joint output.

This CMO is : **Active**

Question for completing the CPT: For each combination of the states of the input nodes, Knowledge, Culture and Trust (eg Medium, High, High states), what is the probability that the state of the Price increases change management option with respect to modifying peak demand be in a High, Low or Nil state? (This question is to be answered remembering that it is not asking the probability of the likely impact of this item given the states of the imput nodes; the impact of this item is addressed with an impact measure in a later sheet.)

Change Management Option	**CMO - Price increases**											
Household	Using segment distributions for Strategy for Queensland											
Knowledge	High				Medium				Low			
Culture	High		Low		High		Low		High		Low	
Trust	High	Low	High	Low	High	Low	High	Low	High	Low	High	Low
High	0.95	0.70	0.80	0.60	0.80	0.60	0.70	0.50	0.60	0.20	0.10	0.00
Low	0.05	0.20	0.20	0.30	0.20	0.30	0.20	0.25	0.30	0.50	0.20	0.10
Nil	0.00	0.10	0.00	0.10	0.00	0.10	0.10	0.25	0.10	0.30	0.70	0.90
	1.00	1.00	1.00	1.00	1.00	1.00	1.00	1.00	1.00	1.00	1.00	1.00

This CMO is : **Active**

Question for completing the CPT: For each combination of the states of the input nodes, Knowledge, Culture and Trust (eg Medium, High, High states), what is the probability that the state of the Appliances (Mininimum performance standards) change management option with respect to modifying peak demand be in a High, Low or Nil state? (This question is to be answered remembering that it is not asking the probability of the likely impact of this item given the states of the input nodes; the impact of this item is addressed with an impact measure in a later sheet.) *It will be noted that the state has been set to High for all conditions as it is a set condition.*

Change Management Option	**CMO - Appliances (Minimum performance standards)**											
Household	Using segment distributions for Strategy for Queensland											
Knowledge	High				Medium				Low			
Culture	High		Low		High		Low		High		Low	
Trust	High	Low	High	Low	High	Low	High	Low	High	Low	High	Low
High	1.00	1.00	1.00	1.00	1.00	1.00	1.00	1.00	1.00	1.00	1.00	1.00
Low												
Nil	0.00	0.00	0.00	0.00	0.00	0.00	0.00	0.00	0.00	0.00	0.00	0.00
	1.00	1.00	1.00	1.00	1.00	1.00	1.00	1.00	1.00	1.00	1.00	1.00

This CMO is : **Active**

Question for completing the CPT: A purchase question. What is the likelihood of installing insulation if a person has () Knowledge, () Culture, and () Trust.

Change Management Option	**CMO - Capital spend - Insulation.** Impacts cooling and heating appliance use (calculated for the House in the external model).											
Household	Using segment distributions for Strategy for Queensland											
Knowledge	High				Medium				Low			
Culture	High		Low		High		Low		High		Low	
Trust	High	Low	High	Low	High	Low	High	Low	High	Low	High	Low
High	0.65	0.60	0.10	0.05	0.60	0.55	0.08	0.09	0.02	0.02	0.00	0.00
Low	0.20	0.20	0.40	0.25	0.25	0.30	0.30	0.19	0.08	0.08	0.06	0.00
Nil	0.15	0.20	0.50	0.70	0.15	0.15	0.62	0.72	0.90	0.90	0.94	1.00
	1.00	1.00	1.00	1.00	1.00	1.00	1.00	1.00	1.00	1.00	1.00	1.00

This CMO is : **Inactive**

Question for completing the CPT: A purchase question. What is the likelihood of purchasing PV if a person has () Knowledge, () Culture, and () Trust.

Change Management Option	**CMO - Capital spend - Photovoltaics.** Impacts cooling and heating appliance use (calculated for the House in the external model).											
Household	Using segment distributions for Strategy for Queensland											
Knowledge	High				Medium				Low			
Culture	High		Low		High		Low		High		Low	
Trust	High	Low	High	Low	High	Low	High	Low	High	Low	High	Low
High	0.05	0.05	0.01	0.00	0.05	0.04	0.01	0.01	0.00	0.00	0.00	0.00
Low	0.10	0.05	0.08	0.05	0.10	0.11	0.04	0.01	0.05	0.05	0.01	0.00
Nil	0.85	0.90	0.91	0.95	0.85	0.85	0.95	0.98	0.95	0.95	0.99	1.00
	1.00	1.00	1.00	1.00	1.00	1.00	1.00	1.00	1.00	1.00	1.00	1.00

Appliance peak demand usage (Household)

Strategic Action Clusters

Acknowledgement & recognition	'Active Elders'	☑
	'Beginnings'	☐
	'Boomer Barons'	☐
	'Cash & Careers'	☐
	'Flush Families'	☐
	'Gen X Parents'	☐
	'Gen X Singles'	☐
	'Golden Years'	☑
	'Leisure Buffs'	☑
	'Mature Wealth'	☐
	'Modest Means'	☐
	'Our Turn'	☑
	'Taking Hold'	☐
	'Transition Blues'	☐

Price increase	'Active Elders'	☑
	'Beginnings'	☑
	'Boomer Barons'	☑
	'Cash & Careers'	☑
	'Flush Families'	☑
	'Gen X Parents'	☑
	'Gen X Singles'	☑
	'Golden Years'	☑
	'Leisure Buffs'	☑
	'Mature Wealth'	☑
	'Modest Means'	☑
	'Our Turn'	☑
	'Taking Hold'	☑
	'Transition Blues'	☑

Customer education & engagement	'Active Elders'	☐
	'Beginnings'	☑
	'Boomer Barons'	☐
	'Cash & Careers'	☑
	'Flush Families'	☑
	'Gen X Parents'	☑
	'Gen X Singles'	☑
	'Golden Years'	☐
	'Leisure Buffs'	☑
	'Mature Wealth'	☐
	'Modest Means'	☑
	'Our Turn'	☐
	'Taking Hold'	☑
	'Transition Blues'	☐

Appliances (minimum performance standards)	'Active Elders'	☐
	'Beginnings'	☐
	'Boomer Barons'	☑
	'Cash & Careers'	☑
	'Flush Families'	☑
	'Gen X Parents'	☐
	'Gen X Singles'	☐
	'Golden Years'	☐
	'Leisure Buffs'	☐
	'Mature Wealth'	☑
	'Modest Means'	☐
	'Our Turn'	☑
	'Taking Hold'	☐
	'Transition Blues'	☐

Off-peak tariffs and managed supply	'Active Elders'	☑
	'Beginnings'	☐
	'Boomer Barons'	☐
	'Cash & Careers'	☐
	'Flush Families'	☑
	'Gen X Parents'	☐
	'Gen X Singles'	☐
	'Golden Years'	☑
	'Leisure Buffs'	☑
	'Mature Wealth'	☑
	'Modest Means'	☑
	'Our Turn'	☑
	'Taking Hold'	☐
	'Transition Blues'	☐

Capital Spend - Insulation	'Active Elders'	☑
	'Beginnings'	☐
	'Boomer Barons'	☑
	'Cash & Careers'	☑
	'Flush Families'	☑
	'Gen X Parents'	☑
	'Gen X Singles'	☑
	'Golden Years'	☐
	'Leisure Buffs'	☑
	'Mature Wealth'	☑
	'Modest Means'	☐
	'Our Turn'	☐
	'Taking Hold'	☐
	'Transition Blues'	☐

Time of use tariffs	'Active Elders'	☐
	'Beginnings'	☐
	'Boomer Barons'	☐
	'Cash & Careers'	☐
	'Flush Families'	☐
	'Gen X Parents'	☐
	'Gen X Singles'	☐
	'Golden Years'	☑
	'Leisure Buffs'	☐
	'Mature Wealth'	☐
	'Modest Means'	☐
	'Our Turn'	☐
	'Taking Hold'	☐
	'Transition Blues'	☐

Capital Spend - Photovoltaics	'Active Elders'	☑
	'Beginnings'	☐
	'Boomer Barons'	☑
	'Cash & Careers'	☐
	'Flush Families'	☑
	'Gen X Parents'	☐
	'Gen X Singles'	☐
	'Golden Years'	☑
	'Leisure Buffs'	☑
	'Mature Wealth'	☑
	'Modest Means'	☐
	'Our Turn'	☐
	'Taking Hold'	☐
	'Transition Blues'	☐
