# Supplementary material for: Systems Modelling of the Socio-Technical Aspects of Residential Electricity Use and Network Peak Demand
Source: PLoS One. 2015 Jul 30;10(7):e0134086. doi: 10.1371/journal.pone.0134086 (PMC4520613; doi:10.1371/journal.pone.0134086)
Supplement: S2 PDF — The conceptual model, Fig 1 in the paper, was developed as the first phase of a larger project and reported in more detail in Buys and colleagues [26]. The figure presents the major components of the system. However, each component consists of sub-networks. In the quantified system, these components produced outputs based on the states of the nodes and the sub-networks that each component contained. There are three broad groupings or domains of the conceptual model components–social, technical and stakeholders–and the sub-networks are described in the following sections along with a description of how the domains are brought together. Further description and definitions of the nodes and other items are presented in Supporting Information (S1, S2, S3 and S4 Tables). (PDF) [file pone.0134086.s002.pdf]

S2 PDF. Description of the model

The conceptual model, Fig. 1 in the paper, was developed as the first phase of a larger project and reported in more detail in Buys and colleagues [26]. The figure presents the major components of the system. However, each component consists of sub-networks. In the quantified system, these components produced outputs based on the states of the nodes and the sub-networks that each component contained. There are three broad groupings or domains of the conceptual model components – social, technical and stakeholders – and the sub-networks are described in the following sections along with a description of how the domains are brought together. Further description and definitions of the nodes and other items are presented in Tables A – D.

1. Social.

The sub-networks were implemented in the spreadsheet by including the modelled elements in the relevant sheet for the Bayesian network.

The components of the social domain were knowledge, trust, culture, household demographics, propensity to change, environmental sensitivity (context)) and the customer-industry engagement.

The state of Trust was modelled as being the result of the state of its input nodes of trust in energy providers and trust in public institutions. The culture node was built on a sub-network consisting of the influence of public support for peak reduction, public support for renewable sources of energy and mandated standards leading to a culture dimension influencing Propensity to Change. The states of the Knowledge node are influenced by the Trust and Customer-Industry Engagement components shown in Fig. 1 of the paper. Additionally, the states of Knowledge are also influenced by the prior states for Knowledge as shown in Fig. 3.

The Propensity to Change node combines the various, selected change management options (CMOs) in the scenario being investigated with their inputs of Knowledge, Culture and Trust for the demographic targeted by the specific CMO in the locality of interest. The context in which this influence is occurring is taken into account through the Environmental Sensitivity (Context) element of the system, whereby if customers were considered to be more likely to modify their peak demand behaviours due to their awareness and current sensitivity to a need for a reduction, this could be accommodated. The household demographic was determined by the location selected using reported information and the specific CMO. An appropriate demographic grouping for each option was used based on industry information [27].

The Customer-Industry Engagement component bridges the two groupings of the social and stakeholder engagement leading to the CMOs that are put in place. The state of the Customer-Industry Engagement component is determined by the degree of engagement that may be undertaken. It is a result of the probability states of the Education and Engagement nodes, which in turn have the probability states set by the level of interventions selected. For each of these Education and Engagement components, they may be set to be occurring at the Broader and the Local Community levels or at the Household (Individual) level. The interactions producing the probability states are shown in Fig. 3 of the paper.

2. Technical.

The technical components of the model consist of the Physical environment, House and Appliances.

The physical environment was represented by the location selected with its specific demographic (applied in the Social domain), the housing stock and the relative electricity use by the locality in the scenario.

The housing stock was applied for the CMO impacting heating and cooling. It was proposed that the input into the model of the effect of the change in heat load, represented by watts per household reduction, would use a separate model for houses with a house energy rating of three and below built

on a change of the proportion of the housing stock moving up one or two scale ratings. In lieu of detailed modelled data, a wattage reduction estimate was used.

The use of appliances by households during peak periods was developed for Queensland. This had winter and summer components that could be modelled separately. The electricity use for other localities was based on this diversified appliance use and the total energy use in each locality.

3. Change management options.

The stakeholders in managing demand side management of electricity by residential customers are the retail market, government policy and the customers themselves (through Customer-Industry Engagement). In the model the stakeholders and the engagement process resulted in the CMOs that were to be modelled. The options were set based on those defined by an energy industry report [27] and the project working group. The single arrow in the model figure (Fig. 1) from CMO in a BN sense consists of the separate arrows from each of the options selected in a given scenario. The calculations are then made with the separate probability tables for each option in the Propensity to Change node of the BN.

The CMOs (described in S3 Table D) considered in this model were:
Acknowledgement & Recognition
Time of Use Tariffs
Off-Peak Tariffs and Managed Supply
Customer Education & Engagement
Price Increase
Appliances (minimum performance standards)
Capital Spend - Insulation
Capital Spend - Photovoltaics

Provision was made so that a further strategic intervention could be investigated if a user wished. Further description of these options is provided in File S3.

The Customer Education & Engagement option, as well as having a direct effect on peak demand, also interacted with the other options to alter their level of impact based on whether the engagement etc. was at the broader community, local community or individual household level.

4. Bringing it all together.

The combining of the social and technical domains for each of the CMOs developed through the stakeholder domain was done in the Appliance usage node of the model. The calculated proportion of the households in a given state, together with the impact on peak demand for each of those states resulted in a network peak load reduction (or increase) for each CMO which were totalled to give the overall network peak demand reduction of the final node.

5. Refining the model.

The model is presented as a method by which a conceptual systems model combining social, technical and policy issues could be quantified. Further elaboration of the sub-networks and the values used would be possible. A strength of Bayesian networks is that they can be refined as additional information on these components becomes available and this will be used to further develop this systems method for combining the social, technical and CMO dimensions of network peak demand for electricity.
