# Supplementary material for: Systems Modelling of the Socio-Technical Aspects of Residential Electricity Use and Network Peak Demand
Source: PLoS One. 2015 Jul 30;10(7):e0134086. doi: 10.1371/journal.pone.0134086 (PMC4520613; doi:10.1371/journal.pone.0134086)
Supplement: S1 Table — Description and definitions of the Nodes of the Bayesian network with a specification of the states of the nodes. These nodes are the Customer-Industry engagement, Knowledge, Trust, Culture, Environmental sensitivity–Context, and Propensity to Change. (PDF) [file pone.0134086.s003.pdf]

S1 Table. Nodes with probabilistic links in the Bayesian network.

Node	Description	Output states
Customer-Industry engagement	Act of engagement of the entity with electricity customers through either imparting particular knowledge or skills and/or through targeted engagement programs aimed at eliciting customer loyalty and advocacy.	*High:-* Specific designed activities aimed at engagement of the entity with electricity customers<br>*Low:-* Minimal designed activities of either education or engagement.
Knowledge	Customers have a combined or an individual understanding of: * peak energy, and/or; * energy costs and benefits, and/or; ; • awareness of demand management energy efficiencies, and/or; • a degree of price consciousness; AND the impact of peak demand on the network and costs of network infrastructure and its ultimate impact on prices.	*High:-* A understanding features of network peak demand<br>*Medium:-* A sound understanding<br>*Low:-* Customers have minimal or no understanding
Trust	Entity (public institutions and/or energy providers) on which one relies FOR INFORMATION OR "POLICY" (reliance on the integrity, strength, ability, surety, etc., of entity; confidence)<br>**Trust in energy providers**<br>Entity (energy providers) on which one relies (reliance on the integrity, strength, ability, surety, etc., of entity; confidence)<br>**Trust in public institutions**<br>Entity (public institutions) on which one relies (reliance on the integrity, strength, ability, surety, etc., of entity; confidence)	*High:-* Acceptance<br>*Low:-* Cynical
Culture	The behaviours and beliefs characteristic of the customers in the area to be modelled as it relates to ***peak energy use***.<br>**Levels**	The customers' characteristic behaviours and beliefs within the area to be modelled<br>*High:-* are conducive to a reduction of peak energy use.<br>*Low:-* are not conducive to a reduction of peak energy use.
Environmental sensitivity - Context	This node covers the context in which the interventions and decisions by a household are being implemented.<br>It captures the Environmental sensitivity of the community etc. impinging on the household and includes societal issues such as climate change, energy pricing, etc.<br>(NB: Environmental sensitivity does not refer to level of awareness of issues relating to the natural environment.)	*High*<br>*Normal*
Propensity to Change	A natural or acquired tendency, inclination, or habit in a person to make desired change of either capital spend or ongoing behaviour change.	Likelihood of making desired changes in either capital spend or ongoing behaviour change.<br>*High:- High*<br>*Low:-* Minimal<br>*Nil:-* No inclination
