# Supplementary material for: Systems Modelling of the Socio-Technical Aspects of Residential Electricity Use and Network Peak Demand
Source: PLoS One. 2015 Jul 30;10(7):e0134086. doi: 10.1371/journal.pone.0134086 (PMC4520613; doi:10.1371/journal.pone.0134086)
Supplement: S2 Table — Description of the elements of the system which impact the technical aspects of the model. These are the Physical environment, House, Appliances, Appliance usage (Household) nodes and their states. (PDF) [file pone.0134086.s004.pdf]

S2 Table. Nodes of the network which specify characteristics impacting on the model

Physical environment	The physical environment specifies the location with the modelled characteristics of number of households, housing profile and climatic and weather extremes.
House	The House node in the model provides for the construction, type, size and star rating of the houses in the given location. This interacts with the Physical Environment to give a heat load for cooling and heating. A change in the star ratings of the houses, together with behavioural changes, such as setting the thermostat at a higher temperature and of not turning the air conditioner on at certain times will interact with the basic house aspects in a location. Although the energy demand of a given (initial) housing profile impacts on the appliances node, the data is collated in the sheet for the House node as it is imported from other modelling but relates to the House profile.
Appliances	A total figure for diversified peak demand usage is derived by estimating the likely appliance energy consumption for cleaning, cooking, living, entertainment and office/work.<br>This usage is specified for Queensland. The figures for Townsville, Toowoomba and any Hotspot are calculated from the Queensland figure.
Appliance usage (Household)	Brings together the propensity to change energy demand behaviour through impact on appliance use.
