# Supplementary material for: Systems Modelling of the Socio-Technical Aspects of Residential Electricity Use and Network Peak Demand
Source: PLoS One. 2015 Jul 30;10(7):e0134086. doi: 10.1371/journal.pone.0134086 (PMC4520613; doi:10.1371/journal.pone.0134086)
Supplement: S3 Table — Description of the Retail market strategies and the Government policy elements of the system. (PDF) [file pone.0134086.s005.pdf]

S3 Table. Description of nodes that are modelled by their influence on Change Management Options and interventions.

Retail market strategies	A major feature of the reform of the electricity sector was the government policy intervention designed to introduce competition into the supply of retail electricity by reducing the barriers to entry for suppliers, allowing consumers to choose their own supplier and thus, encourage innovation and lessen prices.
Government policy	Federal or state government interventions intentionally or unintentionally designed to directly or indirectly impact or affect consumer demand for energy.<br>PV Solar policy<br>Hot water policy (not used in model)<br>Insulation policy<br>Provide households with ease of access to insulation products or initiatives or the setting of clear standards for new home insulation to ensure all new homes have a high standard of insulation in terms of energy efficiency for warming the house in winter and cooling it in summer.<br>Tariff intervention<br>Establishing a pricing structure that is the most effective in reducing or shifting demand during peak periods.
