# Supplementary material for: Systems Modelling of the Socio-Technical Aspects of Residential Electricity Use and Network Peak Demand
Source: PLoS One. 2015 Jul 30;10(7):e0134086. doi: 10.1371/journal.pone.0134086 (PMC4520613; doi:10.1371/journal.pone.0134086)
Supplement: S4 Table — Description of the Change Management Options: Acknowledgment & Recognition; Time of Use Tariffs; Off-Peak Tariffs and Managed Supply; Customer Education & Engagement; Price; Appliances (minimum performance standards); Capital Spend—Insulation, Capital Spend—Photovoltaics and Other Strategic Interventions. (PDF) [file pone.0134086.s006.pdf]

S4 Table. Change Management Options through retail market, government policy and customer-industry engagement.

**Change management options (CMOs)**	Strategic initiatives designed to change or influence consumer behaviour to reduce or shift electricity demand especially during peak periods.
Acknowledgement & Recognition	Customers with low consumption and low payment risk profiles are acknowledged and provided with positive affirmation for their behaviours.
Time of Use Tariffs	Pricing structures tailored for consumers that are the most economical for them generally and also the most effective at dampening electricity demand during peak times or inducing load-shifting electricity demand away from peak times. For example, retirees could benefit from a suitably structured time of use tariff because they are able to undertake energy consumption activity away from peak times.
Off-Peak Tariffs and Managed Supply	Off-Peak tariffs & Managed Supply. Where appliances are hard wired to off-peak or other methods of managed supply
Customer Education & Engagement	(See entry in Table A)
Price	Is the retail price paid by residential customers. It is an imposed cost that affects the price of electricity, for example, carbon tax, network charges etc.
Appliances (minimum performance standards)	Setting energy efficiency standards for appliances available to Australian and Queensland consumers that ensure all appliances on the market are as energy efficient as possible. Poor performing appliances in terms of energy efficiency are blacklisted and unavailable to Australian or Queensland consumers.
Capital Spend<br>Insulation<br>Photovoltaics	Understanding that there is a need to spend capital to save energy. Provide customers with ease of access to household power management for products or initiatives such as Solar PVs, Household modernisation including insulation, efficient pool pumps, alternative hot water of solar or gas.
Other Strategic Interventions	Any other intervention eg EV, Batteries, HEMS, RUS, BlueGen
