# Supplementary material for: Systems Modelling of the Socio-Technical Aspects of Residential Electricity Use and Network Peak Demand
Source: PLoS One. 2015 Jul 30;10(7):e0134086. doi: 10.1371/journal.pone.0134086 (PMC4520613; doi:10.1371/journal.pone.0134086)
Supplement: S5 Table — Table with values of outputs of the model for the Change Management Option nodes for the CIE inputs having Culture, Trust and Knowledge all High or with Culture Low*, or with Trust Low*, or with Knowledge Low*. (PDF) [file pone.0134086.s007.pdf]

S5 Table. Propensity to Change peak demand behaviour with changes in parent nodes.

Change Management Option		With Culture, Trust and Knowledge all High	Culture Low*	Trust Low*	Knowledge Low*
**Acknowledgement & Recognition**					
	High	0.90	0.30	0.70	0.70
	Low	0.10	0.50	0.20	0.20
	Nil	0.00	0.20	0.10	0.10
**Time of Use Tariffs**					
	High	0.95	0.60	0.70	0.80
	Low	0.05	0.30	0.20	0.20
	Nil	0.00	0.10	0.10	0.00
**Off-Peak Tariffs and Managed Supply**					
	High	0.95	0.80	0.70	0.60
	Low	0.05	0.20	0.20	0.30
	Nil	0.00	0.00	0.10	0.10
**Customer Education & Engagement**					
	High	0.10	0.10	0.10	0.00
	Low	0.20	0.20	0.20	0.00
	Nil	0.70	0.70	0.70	1.00
**Price Increases**					
	High	0.95	0.80	0.70	0.60
	Low	0.05	0.20	0.20	0.30
	Nil	0.00	0.00	0.10	0.10
**Appliances (minimum performance standards)**					
	High	1.00	1.00	1.00	1.00
	Low	0.00	0.00	0.00	0.00
	Nil	0.00	0.00	0.00	0.00
**Capital Spend – Insulation**					
	High	0.65	0.01	0.05	0.00
	Low	0.20	0.08	0.05	0.05
	Nil	0.15	0.91	0.90	0.95

*States of the other two nodes set to High.
