# Supplementary material for: Systems Modelling of the Socio-Technical Aspects of Residential Electricity Use and Network Peak Demand
Source: PLoS One. 2015 Jul 30;10(7):e0134086. doi: 10.1371/journal.pone.0134086 (PMC4520613; doi:10.1371/journal.pone.0134086)
Supplement: S6 Table — Output from model for the Impact on electricity demand of households when they were in a High, Low or Nil state of Propensity to Change. Proportion or wattage impact on peak demand that will be reduced for each state. The data indicates, for example, 0.10 is a 10% reduction in peak demand and 280 watt reduction is from a reduction in heat load. (PDF) [file pone.0134086.s008.pdf]

S6 Table. Impact on electricity demand of households being in a High, Low or Nil state of Propensity to Change

	State of Propensity to Change		
**Change Management Option**	**High**	**Low**	**Nil**
Acknowledgement & Recognition	0.10	0.05	0.00
Time of Use Tariffs	0.10	0.05	0.00
Off-Peak Tariffs and Managed Supply	0.10	0.05	0.00
Customer Education & Engagement	0.035	0.01	0.00
Price Increases	0.05	0.02	0.00
Appliances (minimum performance standards)	0.01	0.00	0.00
Capital spend – Insulation			
Cooling (watts reduction)	280	180	0
Heating (watts reduction)	190	140	0

Proportion or wattage impact on peak demand that will be reduced for each state.
The data indicates, for example, 0.10 is a 10% reduction in peak demand
and 280 watt reduction is from a reduction in heat load.
