# Supplementary material for: Systems Modelling of the Socio-Technical Aspects of Residential Electricity Use and Network Peak Demand
Source: PLoS One. 2015 Jul 30;10(7):e0134086. doi: 10.1371/journal.pone.0134086 (PMC4520613; doi:10.1371/journal.pone.0134086)
Supplement: S7 Table — Table with values used in the modelling. The values are the uncertainty applied to impact on peak demand for High and Low states of Propensity to Change. Improvements in these values could be obtained for future iterations of the model development. (PDF) [file pone.0134086.s009.pdf]

S7 Table. Uncertainty applied to impact on peak demand for High and Low states of Propensity to Change

Change Management Option	High	Low
Acknowledgement & Recognition	10.0%	10.0%
Time of Use Tariffs	10.0%	10.0%
Off-Peak Tariffs and Managed Supply	5.0%	5.0%
Customer Education & Engagement	20.0%	20.0%
Price Increases	5.0%	5.0%
Appliances (minimum performance standards)	5.0%	5.0%
Capital Spend – Insulation	20.0%	20.0%
